# Longitudinal Body Composition Identifies Hepatocellular Carcinoma With Cachexia Following Combined Immunotherapy and Target Therapy (CHANCE2213)

**DOI:** 10.1002/jcsm.13615

**Published:** 2024-11-27

**Authors:** Zhi‐Cheng Jin, Jia‐Wei Zhou, Jian‐Jian Chen, Rong Ding, Bernhard Scheiner, Si‐Na Wang, Hai‐Liang Li, Qing‐Xia Shen, Qing‐Yun Lu, Yi Liu, Wei‐Hua Zhang, Biao Luo, Hai‐Bin Shi, Ming Huang, Ye‐Ming Wu, Chun‐Wang Yuan, Ming‐Sheng Huang, Jia‐Ping Li, Jian‐Bing Wu, Xiao‐Li Zhu, Bin‐Yan Zhong, Hai‐Feng Zhou, Yu‐Qing Wang, Shan‐Zhi Gu, Zhi‐Yi Peng, Chuan‐Sheng Zheng, Rui‐Bao Liu, Guo‐Hui Xu, Wei‐Zhu Yang, Ai‐Bing Xu, Dong‐Fang Liu, Xiaolong Qi, Yee Hui Yeo, Hai‐Dong Zhu, Yang Zhao, David J. Pinato, Fanpu Ji, Gao‐Jun Teng

**Affiliations:** ^1^ Center of Interventional Radiology and Vascular Surgery, Department of Radiology, Zhongda Hospital, Medical School Southeast University Nanjing China; ^2^ Basic Medicine Research and Innovation Center of Ministry of Education, Zhongda Hospital, National Innovation Platform for Integration of Medical Engineering Education (NMEE) (Southeast University), State Key Laboratory of Digital Medical Engineering Southeast University Nanjing China; ^3^ Department of Health Statistics, School of Public Health Chongqing Medical University Chongqing China; ^4^ Department of Biostatistics, School of Public Health Nanjing Medical University Nanjing China; ^5^ Department of Minimally Invasive Interventional Medicine Yunnan Cancer Hospital, The Third Affiliated Hospital of Kunming Medical University Kunming China; ^6^ Division of Gastroenterology and Hepatology, Department of Internal Medicine III Medical University of Vienna Vienna Austria; ^7^ Department of Surgery and Cancer, Imperial College London Hammersmith Hospital London UK; ^8^ Department of Minimally Invasive Intervention The Affiliated Cancer Hospital of Zhengzhou University Zhengzhou China; ^9^ Department of Oncology, Zhongda Hospital, Medical School Southeast University Nanjing China; ^10^ Department of Infectious Diseases The Second Affiliated Hospital of Xi'an Jiaotong University, Xi'an China; ^11^ Department of Interventional Radiology The First Affiliated Hospital of Nanjing Medical University Nanjing China; ^12^ Center of Interventional Oncology and Liver Diseases Beijing Youan Hospital, Capital Medical University Beijing China; ^13^ Department of Interventional Radiology, the Third Affiliated Hospital Sun Yat‐sen University Guangzhou China; ^14^ Department of Interventional Oncology The First Affiliated Hospital of Sun Yat‐sen University Guangzhou China; ^15^ Department of Oncology The Second Affiliated Hospital of Nanchang University Nanchang China; ^16^ Department of Interventional Radiology, The First Affiliated Hospital of Soochow University Soochow University Suzhou China; ^17^ Interventional Department Hunan Provincial Tumor Hospital Changsha China; ^18^ Hepatobiliary and Pancreatic Interventional Treatment Center, Division of Hepatobiliary and Pancreatic Surgery, The First Affiliated Hospital Zhejiang University School of Medicine Hangzhou China; ^19^ Department of Radiology, Union Hospital, Tongji Medical College Huazhong University of Science and Technology Wuhan China; ^20^ Department of Interventional Radiology The Tumor Hospital of Harbin Medical University Harbin China; ^21^ Department of Interventional Radiology Sichuan Cancer Hospital and Institute Chengdu China; ^22^ Department of Interventional Radiology Union Hospital of Fujian Medical University Fuzhou China; ^23^ Department of Interventional Therapy Nantong Tumor Hospital Nantong China; ^24^ Center of Portal Hypertension, Department of Radiology, Zhongda Hospital, Medical School Southeast University Nanjing China; ^25^ Karsh Division of Gastroenterology and Hepatology, Department of Medicine Cedars‐Sinai Medical Center Los Angeles CA USA; ^26^ Division of Oncology, Department of Translational Medicine University of Piemonte Orientale “A. Avogadro” Novara Italy; ^27^ Key Laboratory of Environment and Genes Related to Diseases, Xi'an Jiaotong University Ministry of Education of China Xi'an China

**Keywords:** body composition, cachexia, hepatocellular carcinoma, immunotherapy, longitudinal trajectory, molecular targeted therapy

## Abstract

**Background:**

Cancer cachexia can impact prognosis, cause resistance to anticancer treatments and affect the tolerability of treatments. This study aims to identify hepatocellular carcinoma (HCC) with cachexia by characterizing longitudinal body composition (bc) trajectories.

**Methods:**

This longitudinal, multicentre cohort study included unresectable HCC patients treated with first‐line programmed death‐(ligand)1 inhibitors plus anti‐vascular endothelial growth factor antibody/tyrosine kinase inhibitors between 01/2018–12/2022. bc measurements including skeletal muscle mass (SMM) and total adipose tissue area (TATA) were evaluated by computed tomography at the third lumbar vertebra at baseline and follow‐up imaging. Unsupervised latent class growth mixed models were applied to distinguish potential longitudinal SMM and TATA trajectories for identifying cachexia. The primary study endpoint was overall survival (OS), with secondary endpoints including progression‐free survival (PFS), objective response rate (ORR) and safety. Multiple Cox proportional hazards models were used to calculate adjusted hazard ratios (HRs) for survival.

**Results:**

A total of 411 patients with 2138 time‐point measurements were included. The median age was 56 years, and 50 (12.2%) patients were female. Two distinct trajectories were identified for SMM and TATA: sharp‐falling and stable. SMM sharply declined in 58 patients (14.1%) and TATA in 71 of 406 patients (17.5%) with significant worse OS (for SMM, 17.0 vs. 24.9 months; *p* < 0.001; HR = 0.59; for TATA, 15.3 vs. 25.1 months; *p* < 0.001; HR = 0.44). Patients were categorized into three phases based on trajectories: pre‐cachexia (SMM and TATA stable, *n* = 299, 73.6%), cachexia (SMM or TATA sharp‐falling, *n* = 86, 21.2%) and refractory cachexia (SMM and TATA sharp‐falling, *n* = 21, 5.2%). Patients with refractory cachexia exhibited the worst OS, PFS and ORR, followed by those with cachexia. The median OS was 11.5 months for refractory cachexia, 17.7 for cachexia and 26.0 for pre‐cachexia; median PFS was 6.0, 7.9 and 10.9 months, respectively, with ORR of 4.8%, 39.5% and 54.2%, respectively (all *p*s < 0.001). Multivariable Cox analysis identified refractory cachexia as an independent risk factor for both OS (HR = 3.31; *p* < 0.001) and PFS (HR = 2.94; *p* < 0.001), with cachexia also showing significant impacts. Grade 3–4 adverse events were higher in patients with refractory cachexia (23.8%) and cachexia (8.1%) compared with pre‐cachexia (6.0%; *p* = 0.010).

**Conclusions:**

HCC patients with cachexia and refractory cachexia were identified by longitudinal bc trajectories. Falling trajectories of bc identified refractory cachexia patients with worst response, survival and poor tolerability from systemic therapy combinations.

**Trial Registration:**

ClinicalTrials.gov identifier: NCT05278195

## 1 Introduction

Hepatocellular carcinoma (HCC) is the sixth most frequently diagnosed cancer and the second leading cause of cancer‐related deaths worldwide [1]. The success of the IMbrave150 trial using the combination of atezolizumab plus bevacizumab confirmed the efficacy of immunotherapy combinations for HCC [2]. Therefore, systemic treatments with programmed death‐(ligand)1 (PD‐[L]1) inhibitors plus anti‐vascular endothelial growth factor (anti‐VEGF) antibody/tyrosine kinase inhibitors (TKIs)/anti‐CTLA4 antibodies are recommended as first‐line treatment for advanced HCC [3, 4]. However, the proportion of patients who respond to immunotherapy combinations remains modest (approximately one‐third) [2, 5, 6, 7].

Cancer cachexia, a systemic wasting condition, is characterized by the ongoing loss of adipose tissue and skeletal muscle mass (SMM) and contributes to significant morbidity and mortality [8, 9]. It is considered to exist along a continuum with three phases (pre‐cachexia, cachexia and refractory cachexia) according to the international consensus [8, 10]. Patients are classified as having cachexia if they exhibit more than a 5% loss of body weight over the past 6 months, or a body mass index (BMI) < 20 kg/m^2^ with ongoing > 2% weight loss or depletion of muscle mass and >2% weight loss and have not progressed to the refractory stage [8, 10]. Refractory cachexia has been conceptualized as a clinically resistant catabolic state characterized by deteriorated performance status, progressive cancer, resistance to anticancer treatment and a limited life expectancy; nonetheless, there is a lack of consensus regarding the diagnostic criteria for refractory cachexia [10].

Computed tomography (CT)‐derived measurements of body composition (BC) are routinely used to monitor and assess cachexia in clinical settings [10, 11]. Previous studies have explored the role of CT‐based bc measurements in cancer patients in large prospective cohorts or real‐world settings, including those with HCC [12, 13]. However, most studies focus on baseline bc parameters, neglecting longitudinal changes in bc throughout the follow‐up period [14, 15]. Patients may develop cachexia without obvious clinical symptoms, leading to poorer efficacy and tolerability outcomes. Given that the bc in cancer patients exhibits a relatively intricate alteration, bc measurement dynamic changes over time might reveal more informative associations between cachexia and prognosis, which a single assessment fails to capture.

In this longitudinal study, the primary aim was to identify unresectable HCC patients with cachexia by characterizing longitudinal bc measurements trajectories in a large real‐world setting. Our secondary aims were to examine the impact of these trajectories on clinical efficacy and tolerability in patients undergoing first‐line treatment with PD‐(L)1 inhibitors plus anti‐VEGF antibody/TKIs.

## 2 Materials and Methods

The data for this study were derived from the nationwide, multicentre registry database entitled ‘Chinese Liver Cancer Clinical Study Alliance (CHANCE)’ sponsored by the Chinese College of Interventionalists, as detailed in prior publications [16, 17, 18]. Only deidentified data were recorded in a central repository. The study followed the Declaration of Helsinki guidelines and the Strengthening the Reporting of Observational Studies in Epidemiology (STROBE) reporting guideline.

Our study was approved by the ethics committee of Zhongda Hospital, Southeast University (2021ZDSYLL179‐P01), and study protocol was approved by the institutional review boards of participating centres. The need for informed consent was waived by the institutional review board due to the retrospective nature of this study.

### 2.1 Patient Criteria

Between January 2018 and December 2022, all consecutive HCC patients treated with first‐line PD‐(L)1 inhibitor plus anti‐VEGF antibody/TKIs were retrospectively screened from 17 tertiary hospitals. Eligible patients were 18 years of age or older and had histologically, cytologically or clinically confirmed diagnoses of unresectable HCC according to guidelines [19, 20, 21]. The presence of at least one measurable intrahepatic lesion was required. Patients were included if baseline CT images within 3 months before treatment initiation and at least two follow‐up CT images were available.

The exclusion criteria were as follows: (1) previous use of systemic treatment (PD‐(L)1 inhibitors, TKIs or anti‐VEGF antibody); (2) poor baseline CT image quality or severe artefacts; (3) Eastern Cooperative Oncology Group (ECOG) performance status ≥ 2; (4) Child–Pugh grade C or presence of uncontrollable ascites or hepatic encephalopathy; (5) undergone surgery or transplant within 12 months before treatment initiation, or during the follow‐up process; (6) currently had other malignancies or a history of other malignancies in addition to HCC; (7) patients with incomplete follow‐up clinical, or radiological data.

### 2.2 Treatment and Assessment

Individual treatment choices regarding PD‐(L)1 inhibitors and anti‐VEGF antibody/TKIs were in accordance to local practice and guidelines in China [22, 23], involving a comprehensive consideration of the patient's profile, financial burden and patient choice. Transarterial chemoembolization was performed either concurrently, or within a 3‐month window before or after, the administration of PD‐(L)1 inhibitors and anti‐VEGF antibody/TKIs, in order to achieving significant local disease control. Multidisciplinary teams play a crucial role in making these personalized treatment recommendations.

All first‐line PD‐(L)1 inhibitors and anti‐VEGF antibody/TKIs were administrated in accordance with the recommended dose and frequency. Systemic treatment continued until disease progression or unacceptable toxicity. The detailed treatment protocol is described in Appendix S1 and Tables S1 and S2.

Follow‐up visits included regular assessments of vital signs, clinical symptoms, treatment‐related AEs (TRAEs), laboratory testing and radiological examinations by contrast‐enhanced CT or magnetic resonance imaging. The interval of the follow‐up was 6–9 weeks. Tumour response was assessed by two independent radiologists as per the Response Evaluation Criteria in Solid Tumors Version 1.1 (RECIST v1.1). At each visit, TRAEs were recorded and assessed per the National Cancer Institute Common Terminology Criteria for Adverse Events version 5.0 (NCI‐CTCAE v5.0). Patients were followed up until death or the end of the study (30 May 2023).

### 2.3 CT Protocol

The non‐contrast CT scans were acquired in the transverse plane when available (1995 of 2138 scans [93.3%]) and arterial phase or portal phase scans when non‐contrast CT was unavailable (143 of 2138 scans [6.7%]). Detailed CT imaging protocols are described in Appendix S1.

### 2.4 Measurement of BC Parameters

BC parameters were measured both at baseline and during each subsequent follow‐up throughout the treatment process. Cross‐sectional CT images at the level of the third lumbar vertebra for each patient at each point were analysed by two independent observers (Z.C.J. and H.D.Z., with 5 and 15 years of abdominal imaging experience). Observers were blinded to the patient's clinical data and used SliceOmatic V5.0 software (Rev‐8, Tomovision, Montreal, Quebec, Canada) for analyses. In accordance with previous studies, SMM, subcutaneous adipose tissue and visceral adipose tissue were identified and quantified using Hounsfield unit (HU) thresholds of −29 to 150, −190 to −30 and −150 to −50, respectively. The regions of interest were manually adjusted to conform to the actual boundaries of the muscle and subcutaneous fat. The sum of the relevant tissue area and the average density was automatically calculated by summing tissue pixels and multiplying by pixel surface area. Thereafter, the total adipose tissue area (TATA = subcutaneous adipose tissue + visceral adipose tissue) was calculated.

For baseline bc measurement, SMM and TATA were normalized for height in m^2^ to calculate the skeletal muscle index and total adipose tissue index (TATI). The baseline sarcopenia was determined based on previous studies (L3 skeletal muscle index, ≤ 36.2 cm^2^/m^2^ for men and ≤ 29.6 cm^2^/m^2^ for women) [14]. For total adipose tissue, there are no internationally validated cut‐offs. Therefore, the population was dichotomized into 2 groups: patients with sex‐specific high (above median) TATI and low TATI (equal to or below median) subgroups.

### 2.5 Outcomes

The primary study endpoint was overall survival (OS), defined as the time from the initiation of combination therapy to death from any cause or last follow‐up. The secondary study endpoint was progression‐free survival (PFS), objective response rate (ORR) and safety.

PFS was defined as the time from the initiation of combination therapy to first tumour progression, death from any cause, censoring or the end of follow‐up. ORR was defined as the proportion of patients exhibiting a confirmed complete response or partial response. Safety was monitored from initiation of combination therapy throughout the follow‐up period. The incidence and severity of TRAEs were assessed by using the NCI‐CTCAE v 5.0.

### 2.6 Statistical Analysis

Considering multiple assessments of the bc, we performed the latent class growth mixed model (LCGMM), an unsupervised cluster model, to explore the potential classes of trajectories of SMM and TATA over time. Initially, the class number of trajectories was set as 1 to generate the starting value. The bc measurements, including SMM and TATA, was defined as a function of time with covariates, baseline BMI and sex. The linear, quadratic and cubic polynomial functions were fitted, with the class number of trajectories ranging from 2 to 6 in each model. The best fitting number of trajectories was chosen based on the following criteria: the minimum Bayesian Information Criterion; the mean of posterior probabilities in each class ≥ 0.8; and the size of the minimum class ≥ 5% participants. Finally, the quadratic polynomial function of two classes was chosen with the optimal fitness of the SMM and TATA. The assigned labels were defined according to the graphic patterns for interpretation.

Continuous characteristics among different classes were described with mean and standard deviation or median and interquartile range for normal distributed or non‐normal distributed variables and compared with Student's *t*‐test or Wilcoxon Mann–Whitney test, respectively. Count and proportions were reported for categorical variables. The comparison was performed with the chi‐squared test or Fisher's exact test. The OS and PFS were evaluated with the Kaplan–Meier survival curves and log‐rank test. Median follow‐up time was calculated using the reverse Kaplan–Meier method. The hazard ratios (HRs) among different classes were estimated through the Cox proportional hazards model with appropriate covariate adjustments in different models. The relative importance of each covariate on prognosis was assessed by the chi‐squared proportion test with R package ‘rms’. We assessed adherence to the proportional hazards assumption by plotting smoothed Schoenfeld residuals against time; no violations of the assumption were identified.

Additionally, acknowledging the potential presence of immortal time bias in these longitudinal trajectories, which arose from the trajectory class being contingent on future bc measurements and possibly conditionally on survival, the joint modelling analysis was conducted as a sensitive analysis, to examine the correlation between the longitudinal bc measurements and survival outcomes. The SMM and TATA were involved as time‐dependent exposures, and the joint modelling analysis was performed to explore the association between bc measurements and survival after adjusting for baseline variables (detailed in Appendix S1) [24].

Subgroup analyses were performed for prespecified clinically relevant parameters to explore the potential heterogeneity. The correlation between clinical variables and bc trajectory classes was measured using the Spearman correlation coefficient. A *p*‐value less than 0.05 was considered statistically significant. All statistical analyses were performed in R (version 4.1.2) with the packages ‘lcmm’ and ‘JMbayes2’ for the LCGMM and joint modelling, respectively [24, 25].

## 3 Results

### 3.1 Patient Characteristics

A total of 411 patients with individual measurements at 2138 time‐point were finally included in the study (Figure S1). The median times of bc measurements were 4 (interquartile range [IQR], 3–6) per patient. Hepatitis B virus infection was the primary aetiology of liver disease (77.9%), and 235 patients had advanced stage HCC (57.2%). Baseline characteristics are described in Table 1. The median follow‐up duration for the entire cohort was 24.0 months (IQR, 18.0–31.0).

**TABLE 1:** Patient baseline characteristics.

Characteristics	Total population (*n* = 411)	Skeletal muscle trajectory		*p*	Total adipose tissue trajectory^b^		*p*
		Stable (*n* = 353)	Sharp‐falling (*n* = 58)		Stable (*n* = 335)	Sharp‐falling (*n* = 71)	
Age (years)^a^	56 (49–65)	57 (50–66)	52 (47–59)	0.005	56 (49–65)	55 (51–63)	0.612
Sex				0.123			0.668
Female	50 (12.2)	47 (13.3)	3 (5.2)		42 (12.5)	7 (9.9)	
Male	361 (87.8)	306 (86.7)	55 (94.8)		293 (87.5)	64 (90.1)	
BMI	22.1 (20.2–24.2)	22.1 (20.4–24.2)	22.1 (19.3–24.8)	0.718	22.0 (20.2–24.1)	22.7 (21.3–24.7)	0.027
ECOG PS				0.682			0.246
0	310 (75.4)	268 (75.9)	42 (72.4)		256 (76.4)	49 (69.0)	
1	101 (24.6)	85 (24.1)	16 (27.6)		79 (23.6)	22 (31.0)	
Aetiology				> 0.999			0.619
HBV	320 (77.9)	275 (77.9)	45 (77.6)		262 (78.2)	53 (74.6)	
Others	91 (22.1)	78 (22.1)	13 (22.4)		73 (21.8)	18 (25.4)	
Cirrhosis				0.483			0.998
Absent	126 (30.7)	111 (31.4)	15 (25.9)		102 (30.4)	21 (29.6)	
Present	285 (69.3)	242 (68.6)	43 (74.1)		233 (69.6)	50 (70.4)	
Child–Pugh class				0.749			0.718
A	363 (88.3)	313 (88.7)	50 (86.2)		294 (87.8)	64 (90.1)	
B	48 (11.7)	40 (11.3)	8 (13.8)		41 (12.2)	7 (9.9)	
BCLC stage				0.014			0.794
A	45 (10.9)	38 (10.8)	7 (12.1)		37 (11.0)	8 (11.3)	
B	131 (31.9)	122 (34.6)	9 (15.5)		108 (32.2)	20 (28.2)	
C	235 (57.2)	193 (54.7)	42 (72.4)		190 (56.7)	43 (60.6)	
Up‐to‐seven				0.631			0.946
Within	134 (32.6)	113 (32.0)	21 (36.2)		109 (32.5)	24 (33.8)	
Beyond	277 (67.4)	240 (68.0)	37 (63.8)		226 (67.5)	47 (66.2)	
Macrovascular invasion				0.020			0.662
Absent	245 (59.6)	219 (62.0)	26 (44.8)		201 (60.0)	40 (56.3)	
Present	166 (40.4)	134 (38.0)	32 (55.2)		134 (40.0)	31 (43.7)	
Extrahepatic spread				0.042			0.500
Absent	278 (67.6)	246 (69.7)	32 (55.2)		229 (68.4)	45 (63.4)	
Present	133 (32.4)	107 (30.3)	26 (44.8)		106 (31.6)	26 (36.6)	
Serum AFP level				> 0.999			0.837
≤ 400	239 (58.7)	205 (58.7)	34 (58.6)		197 (59.3)	40 (57.1)	
> 400	168 (41.3)	144 (41.3)	24 (41.4)		135 (40.7)	30 (42.9)	

*Note:* Except where indicated, data are number (%). Chi‐squared test or Fisher exact test for categorical variables was applied.

Abbreviations: AFP, alpha‐fetoprotein; BCLC, Barcelona Clinic Liver Cancer; BMI, body mass index; ECOG PS, Eastern Cooperative Oncology Group performance status; HBV, hepatitis B virus.

^a^ Data were continuous variables, expressed in median (interquartile range), and were compared by using the Wilcoxon Mann‐Whitney test.

^b^ Five cases were excluded due to unmeasurable visceral adipose tissue.

### 3.2 Identification of Longitudinal Trajectories

**FIGURE 1:**
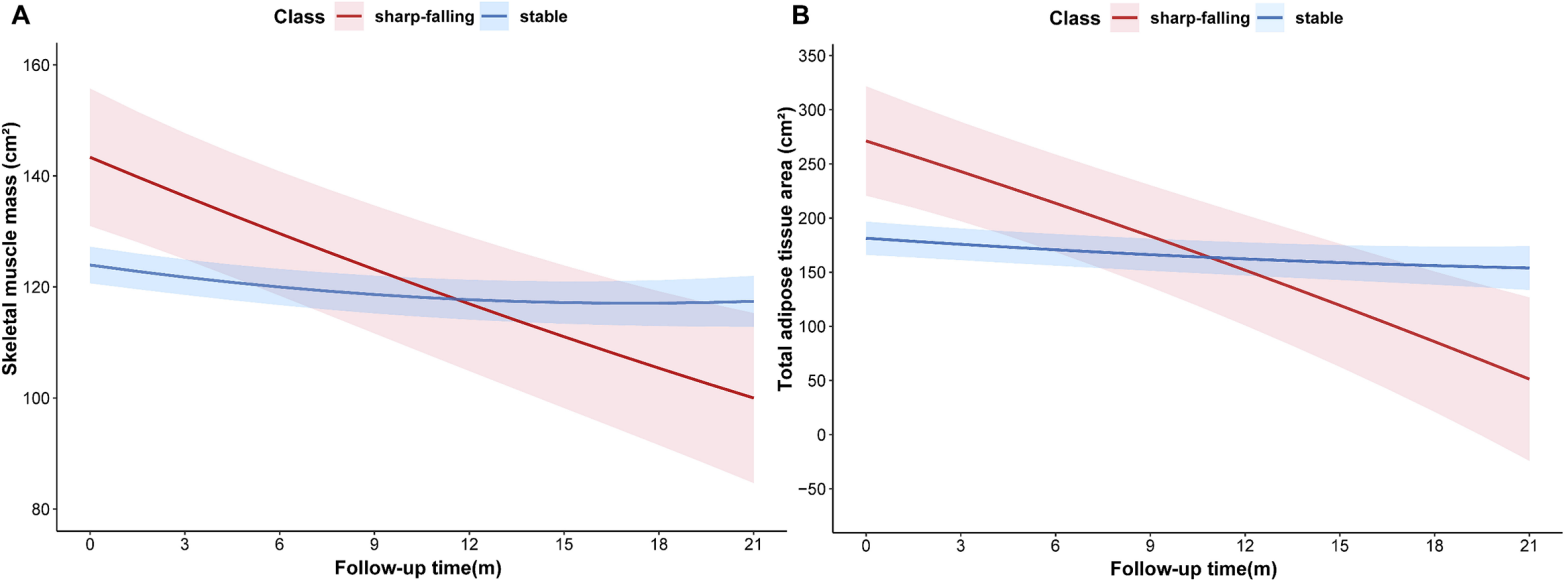
Trajectories for skeletal muscle and adipose tissue in HCC patients after PD‐(L)1 inhibitors and anti‐VEGF antibody/TKIs. Five cases were excluded due to unmeasurable visceral adipose tissue for total adipose tissue area analysis. anti‐VEGF antibody/TKIs, anti‐vascular endothelial growth factor antibody/tyrosine kinase inhibitors; PD‐(L)1 inhibitors, programmed death‐(ligand)1.

Tables S3 and S4 summarize the fitting process for 2 through 6 classes by LCGMM. For SMM, a model of quadratic parameters with two classes provided the optimal fit according to the criteria mentioned above. Thereafter, two distinct trajectories of SMM were identified, labelled as sharp‐falling (14.1%, *n* = 58) and stable (85.9%, *n* = 353). In the sharp‐falling group, SMM declined rapidly from the baseline level over time. Conversely, the stable group maintained a relatively stable SMM. For TATA analysis, five cases were excluded due to unmeasurable visceral adipose tissue. We identified two distinct trajectories of TATA characterized by maintaining a stable tissue area (stable; 335 of 406 [82.5%]) and rapid decline from a level (sharp‐falling; 71 of 406 [17.5%]) throughout the follow‐up. Figure 1 shows the predicted mean trajectory of SMM and TATA.

Compared with the SMM stable class, patients in the sharp‐falling class presented with more advanced stages, accompanied by a higher proportion of vascular invasion and extrahepatic spread (Table 1). For different TATA classes, the BMI of the sharp‐falling class was significantly higher than the stable class.

### 3.3 Identification of Cachexia Staging

Patients were categorized based on their trajectories as follows: Those with stable SMM and TATA were identified as ‘pre‐cachexia’ (*n* = 299, 73.6%); those with either SMM or TATA sharp‐falling class were identified as ‘cachexia’ (*n* = 86, 21.2%); and those with both SMM and TATA sharp‐falling class were identified as ‘refractory cachexia’ (*n* = 21, 5.2%). No significant differences in baseline characteristics were observed across the three stages (Table S5).

### 3.4 Efficacy

**FIGURE 2:**
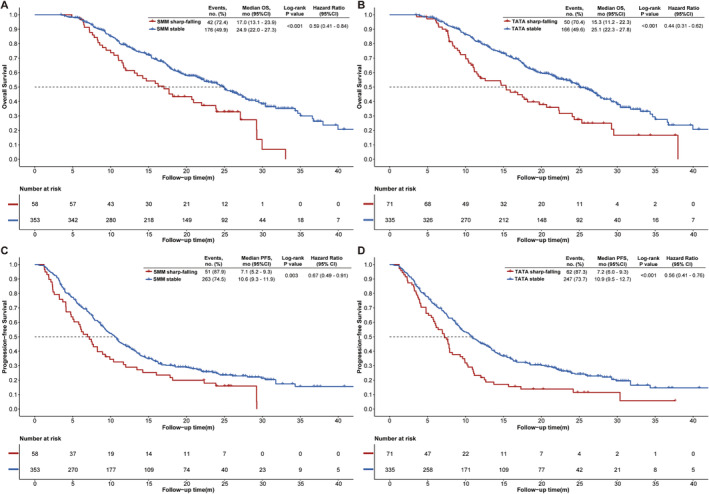
Kaplan–Meier curves of OS and PFS for different trajectory classes. (A) OS curves of SMM trajectory classes in overall patients; (B) OS curves of TATA trajectory classes; (C) PFS curves of SMM trajectory classes in overall patients; (D) PFS curves of TATA trajectory classes; five cases were excluded due to unmeasurable visceral adipose tissue for TATA analysis. OS, overall survival; PFS, progression‐free survival.

During follow‐up, 218 (53.0%) patients died, and the median OS was 23.9 months (95%CI: 20.9–26.3). For SMM trajectory classes, there was a significant difference in median OS between the two classes (stable vs. sharp‐falling, 24.9 months [95%CI: 22.0–27.3] vs. 17.0 months [95%CI: 13.1–23.9]; *p* < 0.001; Figure 2). Multivariable analysis adjusting for potential confounders showed that the SMM stable class was significantly associated with a longer OS (adjusted HR: 0.59, 95%CI: 0.41–0.84; *p* = 0.003) than the SMM sharp‐falling class (Table 2). For TATA trajectory classes, the median OS was 25.1 months [95%CI: 22.3–27.8] and 15.3 months [95%CI: 11.2–22.3] in the stable versus the sharp‐falling class, respectively (*p* < 0.001). Compared with the TATA sharp‐falling class, patients in TATA stable class had a longer OS (adjusted HR: 0.44, 95%CI: 0.31–0.62; *p* < 0.001). Median PFS and ORR also differed significantly among these two classes in both SMM and TATA (detailed in Appendix S1).

**TABLE 2:** Trajectory classes of body composition and hazard ratios.

	Non‐adjusted	*p*	Model 1	*p*	Model 2	*p*	Model 3	*p*
OS analyses								
SMM								
Stable vs. sharp‐falling	0.56 (0.40–0.79)	0.001	0.58 (0.41–0.82)	0.002	0.57 (0.40–0.80)	0.001	0.59 (0.41–0.84)	0.003
TATA^a^								
Stable vs. sharp‐falling	0.52 (0.38–0.71)	< 0.001	0.48 (0.35–0.66)	< 0.001	0.47 (0.33–0.66)	< 0.001	0.44 (0.31–0.62)	< 0.001
PFS analyses								
SMM								
Stable vs. sharp‐falling	0.63 (0.47–0.86)	0.003	0.64 (0.48–0.87)	0.005	0.64 (0.47–0.86)	0.004	0.67 (0.49–0.91)	0.011
TATA^a^								
Stable vs. sharp‐falling	0.60 (0.45–0.79)	< 0.001	0.58 (0.43–0.77)	< 0.001	0.58 (0.43–0.77)	< 0.001	0.55 (0.41–0.75)	< 0.001

*Note:* Data are hazard ratios (95% confidence intervals). Model 1 was adjusted for age, sex and BMI. Model 2 was adjusted for age, sex, BMI and baseline body composition (for skeletal muscle analysis, non‐sarcopenia vs. sarcopenia; for adipose tissue analysis, low TATI vs. high TATI). Model 3 included the following variables: age, sex, BMI, ECOG PS, Child–Pugh class, up‐to‐seven criteria, BCLC stage and baseline body composition (for skeletal muscle analysis, non‐sarcopenia vs. sarcopenia; for adipose tissue analysis, low TATI vs. high TATI).

^a^ Five cases were excluded due to unmeasurable visceral adipose tissue.

Abbreviations: BCLC, Barcelona Clinic Liver Cancer; BMI, body mass index; ECOG PS, Eastern Cooperative Oncology Group performance status; SMM, skeletal muscle mass; TATA, total adipose tissue area; TATI, total adipose tissue index.

**FIGURE 3:**
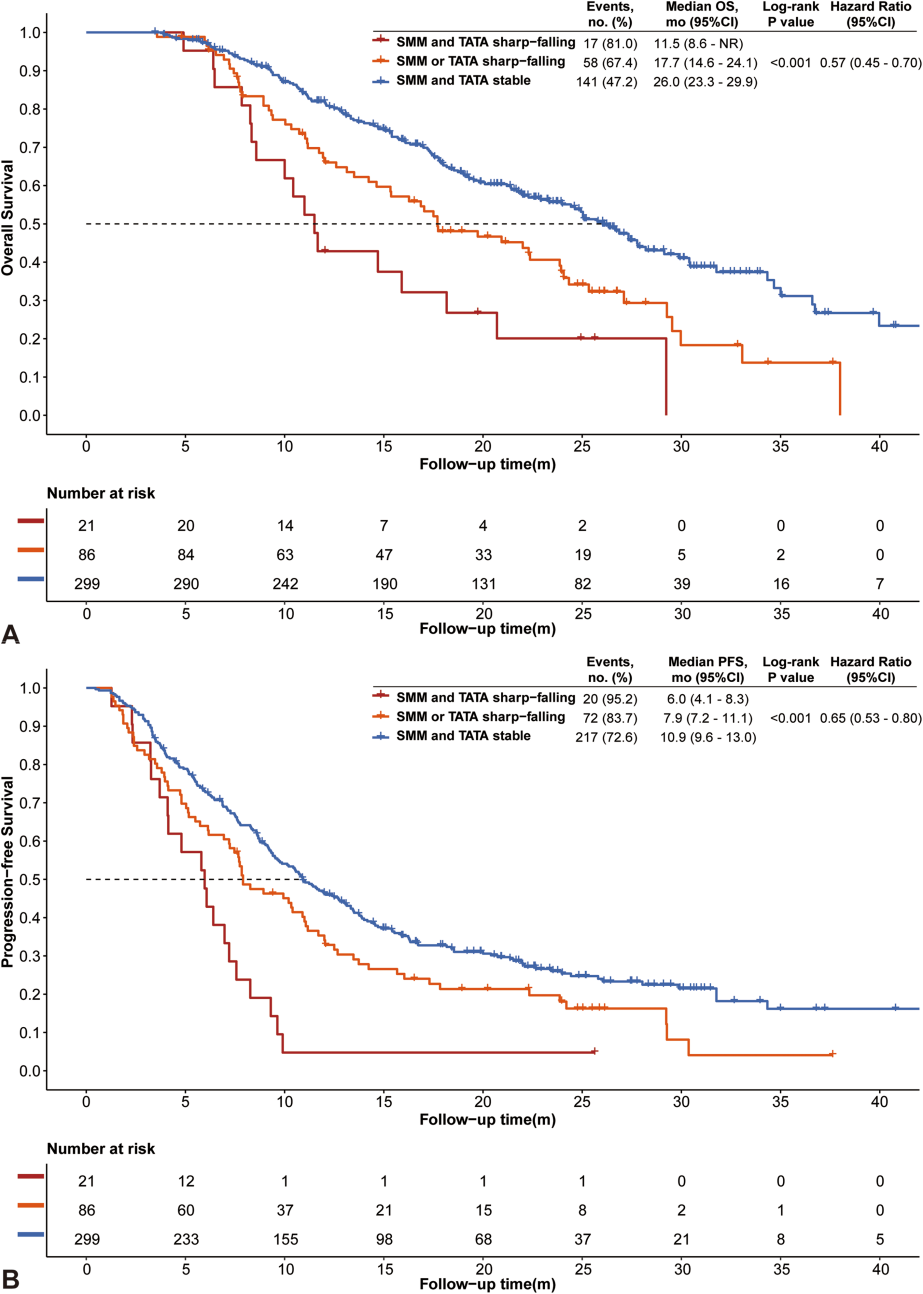
Kaplan–Meier curves of OS (A) and PFS (B) of the presence of cachexia during follow‐up. OS, overall survival; PFS, progression‐free survival; SMM, skeletal muscle mass; TATA, total adipose tissue area.

Patients with refractory cachexia had the worst OS, PFS and ORR, followed by patients with cachexia (median OS, 11.5 [95%CI: 8.6‐NR] vs. 17.7 [95%CI: 14.6–24.1] vs. 26.0 [95%CI: 23.3–29.9] months; *p* < 0.001; median PFS, 6.0 [95%CI: 4.1–8.3] vs. 7.9 [95%CI: 7.2–11.1] vs. 10.9 [95%CI: 9.6–13.0] months; *p* < 0.001; Figure 3; ORR, 4.8% vs. 39.5% vs. 54.2%; *p* < 0.001). Multivariable COX analysis showed that refractory cachexia was an independent risk factor for OS (adjusted HR: 3.31, 95%CI: 1.91–5.75; *p* < 0.001) and for PFS (adjusted HR: 2.94, 95%CI: 1.80–4.81; *p* < 0.001), while similar results were also shown in cachexia (for OS, adjusted HR = 1.86 [95%CI: 1.35–2.57]; for PFS, adjusted HR = 1.37 [95%CI: 1.03–1.81]; Table 3).

**TABLE 3:** Hazard ratios and the presence of cachexia during follow‐up.

	OS analyses				PFS analyses			
	Non‐adjusted hazard ratios	*p*	Adjusted hazard ratios	*p*	Non‐adjusted hazard ratios	*p*	Adjusted hazard ratios	*p*
Pre‐cachexia	1		1		1		1	
Cachexia	1.70 (1.25–2.31)	0.001	1.86 (1.35–2.57)	< 0.001	1.39 (1.07–1.82)	0.015	1.37 (1.03–1.81)	0.028
Refractory cachexia	2.97 (1.79–4.94)	< 0.001	3.31 (1.91–5.75)	< 0.001	2.79 (1.75–4.43)	< 0.001	2.94 (1.80–4.81)	< 0.001

*Note:* Data are hazard ratios (95% CIs). Multivariable Cox model included the following variables: age, sex, BMI, ECOG PS, Child–Pugh class, up‐to‐seven criteria, BCLC stage, baseline skeletal muscle analysis (non‐sarcopenia vs. sarcopenia) and baseline adipose tissue (low TATI vs. high TATI).

Abbreviations: BCLC, Barcelona Clinic Liver Cancer; BMI, body mass index; ECOG PS, Eastern Cooperative Oncology Group performance status; SMM, skeletal muscle mass; TATA, total adipose tissue area; TATI, total adipose tissue.

### 3.5 Adverse Events

Treatment‐emergent AEs were reported by 12 of 21 patients (57.1%) with refractory cachexia, 30 of 86 patients (34.9%) with cachexia and 73 of 299 patients (24.4%) with pre‐cachexia (*p* = 0.002; Table S6). Grade 3 or 4 AEs occurred in 5 patients (23.8%) in refractory cachexia compared with 7 (8.1%) in cachexia and 18 (6.0%) in pre‐cachexia (*p* = 0.010). No grade 5 AEs were observed in the entire study population.

The PD‐(L)1 inhibitors were discontinued due to AEs in 3 patients (14.3%) with refractory cachexia, 3 patients (3.5%) with cachexia and 14 patients (4.7%) with pre‐cachexia (*p* = 0.114). Molecular targeted agents were discontinued in 4 patients (19.0%) with refractory cachexia, 2 patients (2.3%) with cachexia and 15 patients (5.0%) with pre‐cachexia (*p* = 0.008). Dose interruptions of PD‐(L)1 inhibitors were observed in 2 patients (9.5%) with refractory cachexia, 2 patients (2.3%) with cachexia and 13 patients (4.3%) with pre‐cachexia (*p* = 0.324). Dose reduction or interruption of targeted agents was reported by 5 patients (23.8%) with refractory cachexia, 12 patients (14.0%) with cachexia and 32 patients (10.7%) with pre‐cachexia.

### 3.6 Sensitive Analyses and Exploratory Analyses

In sensitive analyses, the multivariable joint model showed that SMM and TATA showed significant association with survival (for SMM, adjusted HR: 0.85, 95% confidence interval [CI]: 0.70–0.94, *p* < 0.001; for TATA, adjusted HR: 0.96, 95%CI: 0.92–0.99, *p* < 0.001; Table S7).

**FIGURE 4:**
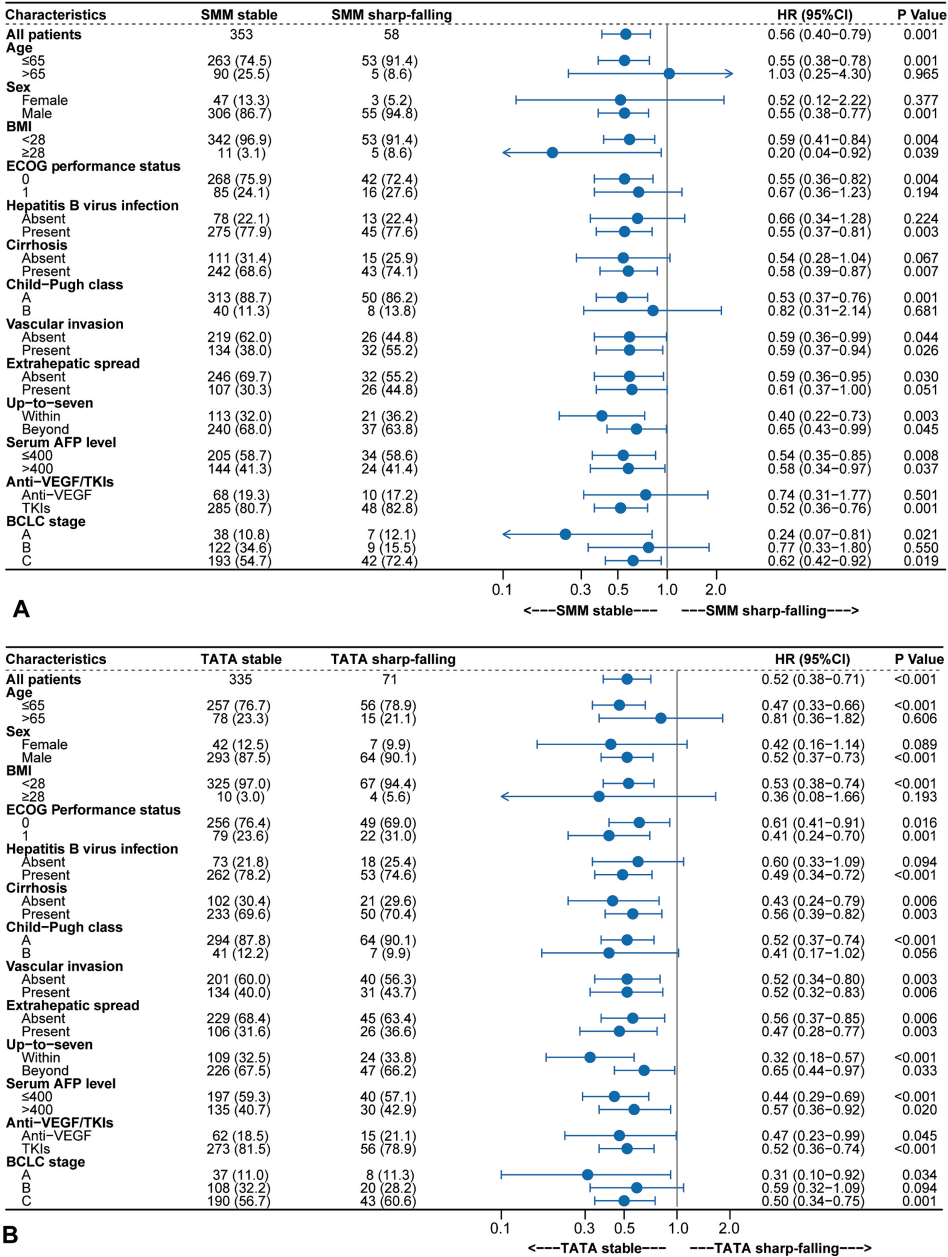
Subgroup analysis of OS for SMM trajectory classes (A) and TATA trajectory classes (B). AFP, alpha‐fetoprotein; BCLC, Barcelona Clinic Liver Cancer; BMI, body mass index; CI, confidence interval; ECOG, Eastern Cooperative Oncology Group; HR, hazard ratio; OS, overall survival; SMM, skeletal muscle mass; TATA, total adipose tissue area.

We conducted analyses comparing survival risk between different trajectories for prespecified clinically relevant parameters. The OS benefits of the SMM or TATA stable class, as shown in Figure 4, were generally consistent across all subgroups compared to the SMM or TATA sharp‐falling class. Similar trends were observed in PFS benefits in these patients (Figures S2 and S3). The correlation was shown by heatmap between clinical variables and longitudinal bc trajectory classes in HCC patients (Figure S4). Longitudinal TATA trajectories were positively correlated with longitudinal SMM trajectories. Venn diagrams show the distribution of patients' longitudinal bc trajectory classes and baseline bc measurements (Figures S5–S7).

Furthermore, we analysed the relative contribution of each risk factor to predict OS and PFS (Figures S8 and S9). The importance of cachexia staging was stronger than all these clinical variables for PFS and OS, except for SMM trajectory group is ranked second for OS. To facilitate clinical practice, a web application (https://bclctm.shinyapps.io/prediction_model_platform/) was constructed to implement cachexia phases categorization of new patients, based on the longitudinal bc measurements.

## 4 Discussion

Cachexia is multifactorial and found to be driven by tumour‐ and inflammatory‐secreted factors, with negative effects on efficacy and tolerability from anti‐cancer therapy [26, 27]. The assessment of cachexia in HCC is particularly challenging due to its cirrhosis background. This pathophysiological process also persists during the entire process of tumour development and progression. CT‐derived bc measurement are easier to monitor longitudinally and may facilitate earlier detection of patients with cachexia [9].

To the best of our knowledge, this is the first study to comprehensively characterize the latent longitudinal trajectories of bc changes. In this study, time‐series imaging‐based data were utilized to identify distinct trajectories among unresectable HCC patients undergoing immunotherapy plus targeted therapy. These trajectories were categorized as either stable or sharp‐falling for skeletal muscle and for total adipose tissue. Our study found significant associations between bc trajectories and OS, PFS and ORR. The effects of trajectories on mortality and progression were also analysed across several subgroups. Despite the potential for immortal time bias in the trajectory analysis, the results from the joint modelling, which was designed to address this bias, indicated elevated bc measurements associated with a more favourable prognosis. This finding aligned with that the sharp‐falling class exhibited a shorter survival period when compared to the stable class.

Importantly, our study found that 21 out of 406 patients (5.2%) displayed a rapid longitudinal decrease in both skeletal muscle and adipose tissue, classified as ‘refractory cachexia’, and 86 patients (21.2%) with skeletal muscle or adipose tissue sharp‐falling class were categorized as ‘cachexia’. These findings align with the reported cachexia prevalence in HCC, where nearly 1 in 4 patients were affected [28]. Approximately 20% of cachexia patients in this study were identified as refractory cachexia, which was not previously reported. These patients were associated with worse survival outcomes and a higher incidence of high‐grade AEs, which is particularly significant in the management of HCC, where preservation of quality of life is integral to the multi‐disciplinary management of these patients [29]. Notably, these patients with presence of cachexia were found during the follow‐up, rather than diagnosed at the time of initiation of combination therapy. These changes in BC are challenging to clinically detect and require the attention of clinicians.

Skeletal muscles, recognized as endocrine organs, perform their biological roles by releasing a diverse array of myokines [30]. The regulation of cytokine secretion during muscle catabolism may induce systemic inflammation and immunosuppression, which are associated with worse outcomes [9, 31]. Several clinical studies have reported significantly shorter survival among baseline sarcopenic patients undergoing immunotherapy [32, 33]. The incidence of immune‐related AEs is similar in sarcopenic and non‐sarcopenic patients. In this study, a subset of patients (14%) with longitudinal SMM sharp‐falling were identified which was ‘rapid sarcopenia developers’. Interestingly, the presence of sarcopenia at baseline did not significantly overlap with those patients of the SMM sharp‐falling class (Figure S5). Correlation analyses revealed that longitudinal sarcopenia is associated with baseline macrovascular invasion and extrahepatic spread, suggesting a more aggressive tumour profile (Figure S4). These findings strongly suggest that SMM sharp‐falling class is an independent risk factor warranting evaluation during dynamic follow‐up.

The prognostic impact of adipose tissue in immunotherapy is complex. Recent preclinical and clinical studies showed an unexpected inverse relationship between obesity and the efficacy of PD‐(L)1 inhibitors, the so‐called ‘obesity paradox’ [34]. Obesity not only leads to tumour progression, immune ageing and PD‐1‐mediated T cell dysfunction but also is linked to enhanced efficacy of PD‐1/PD‐L1 inhibitors [35]. Caan et al. found this phenomenon is better described as a ‘BMI paradox’ [36]. That is, BMI is an inadequate proxy for adiposity, failing to differentiate between muscle and adipose tissue or delineate adipose tissue distribution. A meta‐analysis indicates that overweight/obese males exhibit the strongest survival benefit, while overweight/obese females show no significant benefit [32]. Overweight/obese patients of both sexes were at higher risk of developing high‐grade immune‐related AEs. In this study, the median BMI of the TATA sharp‐falling group was significantly higher than that of the stable group; however, this difference (22.7 vs. 22.0) was not large. Most patients (84.5%, 60/71) in the TATA sharp‐falling group have baseline high adipose tissue (Figure S6). Considering the multifaceted effects of obesity and adipose tissue, further research with a larger sample size is encouraged to validate the findings.

As an observational retrospective study, our results reflect therapeutic decisions made in the so‐called ‘real world’, outside of stringent clinical trial protocols. Indeed, this approach has well‐known limitations and potential bias. First, some important clinical variables such as BMI and liver function parameters were not longitudinally collected in this study. Also, MRI‐derived BC parameters were not computed, leaving to attrition of data for patients who underwent abdomen MRI during follow‐up. These longitudinal clinical and imaging data may reveal more information concerning the relationship between BC changes and fluctuations in liver functional reserve. Second, this study did not capture the cachexia‐related treatment strategies [37]. To date, no validated interventions have been identified according to guidelines [10]. Theoretically, a multimodal lifestyle‐based intervention, such as dietary counselling, nutritional support and exercise, could prevent the progression from pre‐cachexia to cachexia phase and manage cancer cachexia [37]. An interdisciplinary health team with diverse expertise may enhance the quality of cancer care. Third, patients with ≤ 2 follow‐up imaging, representing a group with potentially worse prognosis, were excluded from this study. This may cause the clinical outcomes improved in the whole cohort. However, for latent class modelling of longitudinal data, at least three measurement time points are typically necessary to ensure accurate estimations [38]. Fourth, the current bc trajectories were based on Chinese HCC patients with predominately hepatitis B infection and cirrhosis, posing an issue of generalizability with the wider landscape of HCC.

In conclusion, this study identified distinct trajectories of BC through repeated measurements for HCC patients. Falling trajectories of bc were associated with worse response, survival and poor tolerability to systemic therapy combinations. Moreover, patients with a rapid longitudinal decline in both skeletal muscle and adipose tissue exhibited the worst prognosis and may experience a specified process of ‘refractory cachexia’. These findings offer novel insights into the diversity of cancer cachexia and could pave the way for nutritional interventions to prevent disease progression.

## Ethics Statement

The study was approved by the institutional review board (IRB) of Zhongda Hospital Southeast University approval as well as applicable local laws and regulatory requirements (Ethics Approval ID: 2021ZDSYLL179‐P01). The need for informed consent was waived by the IRB due to the retrospective nature of this study.

## Conflicts of Interest

Author's declaration of personal interests are as follows: Dr. Bernhard Scheiner received grant support from AstraZeneca and Eisai, speaker honoraria from Eisai and travel support from AbbVie, AstraZeneca, Ipsen and Gilead. Dr. David J. Pinato received lecture fees from ViiV Healthcare and Bayer Healthcare and travel expenses from BMS and Bayer Healthcare; consulting fees for Mina Therapeutics, EISAI, Roche, Astra Zeneca, DaVolterra, Exact Sciences, MURSLA, Avamune, BMS, LIfT Biosciences and Starpharma; and received research funding (to institution) from MSD, BMS and GSK. Dr. Fanpu Ji received lecture fees from Gilead Sciences, MSD and Ascletis; he is a consultant for Gilead, MSD. All other authors declare no conflicts of interest.

## Supporting information


**Table S1** Agents administration protocol.
**Table S2.** Number of patients treated with combination therapy.
**Table S3.** LCGMM results of the model fitting process for SMM.
**Table S4.** LCGMM results of the model fitting process for TATA.
**Table S5.** Baseline characteristics of the presence of cachexia during follow‐up.
**Table S6.** Treatment‐emergent AE of the presence of cachexia during follow‐up.
**Table S7.** The results of joint modelling.
**Figure S1.** Flowchart of the study.
**Figure S2.** Subgroup analysis of PFS for longitudinal SMM trajectory.
**Figure S3.** Subgroup analysis of PFS for longitudinal TATA trajectory.
**Figure S4.** Heatmap of the correlation between clinical variables and longitudinal body composition trajectory classes in HCC patients.
**Figure S5.** Venn diagram of longitudinal SMM and baseline skeletal muscle (sarcopenia/non‐sarcopenia) distributions.
**Figure S6.** Venn diagram of longitudinal TATA and baseline TATI distributions.
**Figure S7.** Venn diagram of longitudinal SMM and TATA distributions.
**Figure S8.** The relative importance of each risk factor for OS.
**Figure S9.** The relative importance of each risk factor for PFS.
Reference

**Data S1** Supporting information.
