# Supplementary material for: Longitudinal Body Composition Identifies Hepatocellular Carcinoma With Cachexia Following Combined Immunotherapy and Target Therapy (CHANCE2213)
Source: J Cachexia Sarcopenia Muscle. 2024 Nov 27;15(6):2705–16. doi: 10.1002/jcsm.13615 (PMC11634469; doi:10.1002/jcsm.13615)
Supplement: Supplementary file 1 — Table S1 Agents administration protocol. Table S2. Number of patients treated with combination therapy. Table S3. LCGMM results of the model fitting process for SMM. Table S4. LCGMM results of the model fitting process for TATA. Table S5. Baseline characteristics of the presence of cachexia during follow‐up. Table S6. Treatment‐emergent AE of the presence of cachexia during follow‐up. Table S7. The results of joint modelling. Figure S1. Flowchart of the study. Figure S2. Subgroup analysis of PFS for longitudinal SMM trajectory. Figure S3. Subgroup analysis of PFS for longitudinal TATA trajectory. Figure S4. Heatmap of the correlation between clinical variables and longitudinal body composition trajectory classes in HCC patients. Figure S5. Venn diagram of longitudinal SMM and baseline skeletal muscle (sarcopenia/non‐sarcopenia) distributions. Figure S6. Venn diagram of longitudinal TATA and baseline TATI distributions. Figure S7. Venn diagram of longitudinal SMM and TATA distributions. Figure S8. The relative importance of each risk factor for OS. Figure S9. The relative importance of each risk factor for PFS. Reference [file JCSM-15-2705-s002.docx]

**Supplementary Appendix**

This appendix has been provided by the authors to give readers additional information about their work.

**Content**

[Materials and Methods 3](#_Toc157687852)

[***Computed Tomography Imaging Protocol*** 3](#_Toc157687853)

[***Immune Checkpoints Inhibitors Administration*** 4](#_Toc157687854)

[***Anti-VEGF antibody/tyrosine kinase inhibitors Administration*** 4](#_Toc157687855)

[***Statistical analysis*** 4](#_Toc157687856)

[Results 6](#_Toc157687857)

[***Efficacy*** 6](#_Toc157687858)

[Table S1. Agents Administration Protocol 8](#_Toc157687859)

[Table S2. Number of patients treated with combination therapy 10](#_Toc157687860)

[Table S3. LCGMM results of the model fitting process for SMM 11](#_Toc157687861)

[Table S4. LCGMM results of the model fitting process for TATA 12](#_Toc157687862)

[Table S5. Baseline characteristics of the presence of cachexia during follow-up 13](#_Toc157687863)

[Table S6. Treatment-emergent AE of the presence of cachexia during follow-up 15](#_Toc157687864)

[Table S7. The results of joint modeling 16](#_Toc157687865)

[Figure S1. Flowchart of the study. 17](#_Toc157687866)

[Figure S2. Subgroup analysis of PFS for longitudinal SMM trajectory. 18](#_Toc157687867)

[Figure S3. Subgroup analysis of PFS for longitudinal TATA trajectory. 19](#_Toc157687868)

[Figure S4. Heatmap of the correlation between clinical variables and longitudinal body composition trajectory classes in HCC patients. 20](#_Toc157687869)

[Figure S5. Venn diagram of longitudinal SMM and baseline skeletal muscle (sarcopenia/non-sarcopenia) distributions. 21](#_Toc157687870)

[Figure S6. Venn diagram of longitudinal TATA and baseline TATI distributions. 22](#_Toc157687871)

[Figure S7. Venn diagram of longitudinal SMM and TATA distributions 23](#_Toc157687872)

[Figure S8. The relative importance of each risk factor for OS. 24](#_Toc157687873)

[Figure S9. The relative importance of each risk factor for PFS. 25](#_Toc157687874)

[Reference 26](#_Toc157687875)

Materials and Methods

***Computed Tomography Imaging Protocol***

The non-contrast computed tomography (CT) scans were acquired in the transverse plane when available (1995 of 2138 scans [93.3%]) and arterial phase or portal phase scans when non-contrast CT was unavailable (143 of 2138 scans [6.7%]). The CT scans were using one of the following systems: SIEMENS SOMATOM Definition AS+, GE Revolution CT, SIEMENS SOMATOM Force, SIEMENS SOMATOM Definition Flash, GE LightSpeed VCT, Philips iCT 256, Philips Ingenuity CT, TOSHIBA Aquilion PRIME, Philips IQon - Spectral CT, GE Discovery CT750, SIEMENS SOMATOM Perspective, and UIH uCT 760. The technical parameters for CT imaging were as follows: the tube voltage ranged from 90 to 150 (mode, 120 kVp), the tube current of 60‐600 mAs (mode, 220 mAs), and the section thickness ranged from 1 to 10 mm (mode, 5 mm).

When non-contrast CT was not available and contrast-enhanced CT scans were performed, the contrast agents used were iodixanol (Ultravist 300 or Ultravist 370, Bayer, Germany) and ioversol (Optiray 320 or Optiray 350, Guerbet, France). They were administered intravenously at a dosage of 1.3-1.5 ml per kilogram of body weight and a flow rate of 3.0–4.0 mL/s. Arterial phase scanning was initiated with about 20-30 seconds delay after enhancement of the descending aorta to 100 HU, as measured using a bolus-tracking technique; portal venous and delayed phase images were obtained at 50–60 and 110-120 seconds, respectively, after injection of contrast.

***Immune Checkpoints Inhibitors Administration***

A range of immune checkpoint inhibitors (ICIs) including Atezolizumab, Camrelizumab, Sintilimab, Pembrolizumab, Nivolumab, and Tislelizumab were utilized, upon guidelines and availability in China. The administration of these ICIs strictly adhered to prescribed dosages and frequencies. While dose reduction was not permitted, temporary discontinuation of ICIs due to adverse events (AEs) was acceptable. Patients continued to receive anti-PD-(L)1 agents until either disease progression or unacceptable toxicities.

***Anti-VEGF antibody/tyrosine kinase inhibitors Administration***

Several anti-VEGF antibodies/tyrosine kinase inhibitors (TKIs) including Sorafenib, Lenvatinib, Donafenib, Apatinib, and Bevacizumab or its’ biosimilar, were administered in accordance with prescribed dosages and frequencies. For these agents, dose reduction in response to grade 3 or 4 AEs was allowed, with the exception of Bevacizumab and its biosimilars. Treatment with anti-VEGF antibodies/TKIs was continued until disease progression or unacceptable toxicities.

***Statistical analysis***

In this study, we considered the potential immortal time bias and explored the association between longitudinal changes in BC measurements and survival outcomes. However, the classical approaches such as the linear mixed model (for longitudinal data) and the Cox proportional hazards model (for time-to-event data) do not consider interdependencies between these two data types (longitudinal and time-to-event) simultaneously.^1^ The joint modeling is a powerful method that brings the two data types together simultaneously with two linked sub-models so that we could estimate the association between the longitudinal and time-to-event data. We considered skeletal muscle mass (SMM) and total adipose tissue area (TATA) as time-dependent variables and performed the joint modeling analysis to explore the association between the BC measurements and survival outcomes, after adjusting baseline variables. The linear mixed-effects sub-model was fitted to describe the evolution of BC measurements (SMM and TATA separately) over time, which is objective to immortal time bias, with adjustment for sex and body mass index (BMI). A Cox proportional hazards sub-model was used to screen covariates that may be associated with survival outcomes, such as sex, age, ECOG performance score, BMI, cirrhosis, hepatitis B virus infection, BCLC stage, up-to-seven criteria, vascular invasion, and extrahepatic spread at baseline. The parameter estimation within the multivariate joint modeling framework was performed using the R package “JMbayes2”, and implemented with the Markov Chain Monte Carlo algorithms.

Results

***Efficacy***

For SMM trajectory classes, there was a significant difference in median OS between the two classes (stable vs. sharp-falling, 24.9 months [95%CI: 22.0-27.3] vs. 17.0 months [95%CI: 13.1-23.9]; P < 0.001). Median PFS and ORR also differed significantly among these two classes (for PFS, 10.6 months [95%CI: 9.3-11.9] vs. 7.1 months [95%CI: 5.2-9.3]; P = 0.003; for ORR, 51.3% vs. 29.3%; P = 0.003; Figure 2). Multivariable analysis adjusting for potential confounders showed that the SMM stable class was significantly associated with a longer OS (adjusted HR: 0.59, 95%CI: 0.41-0.84; P = 0.003) and longer PFS (adjusted HR: 0.67, 95%CI: 0.49-0.91; P = 0.011) than the SMM sharp-falling class (Table 2).

For TATA trajectory classes, the median OS was 25.1 months [95%CI: 22.3-27.8] and 15.3 months [95%CI: 11.2-22.3] in the stable versus the sharp-falling class, respectively (P < 0.001). Similar results were also shown for median PFS (10.9 [95%CI: 9.5-12.7] vs. 7.2 [95%CI: 6.0-9.3]; P < 0.001) and ORR (52.8% vs. 28.2%; P < 0.001). Compared with the TATA sharp-falling class, patients in TATA stable class had a longer OS (adjusted HR: 0.44, 95%CI: 0.31-0.62; P < 0.001) and longer PFS (adjusted HR: 0.55, 95%CI: 0.41-0.75; P < 0.001).

***Adverse events***

Treatment-emergent AEs were reported by 26 of 58 patients (44.8%) in SMM sharp-falling class and 92 of 353 patients (26.1%) in SMM stable class (P = 0.006; Table S5). Grade 3 or 4 AEs occurred in 10 patients (17.2%) in SMM sharp-falling class compared with 23 (6.5%) in SMM stable class (P = 0.012). No grade 5 AEs were observed in the entire study population. Treatment-emergent AEs were reported by 29 of 71 patients (40.8%) in the TATA sharp-falling class, and 86 of 335 patients (25.7%) in the TATA stable class (P = 0.015; Table S5). Grade 3 or 4 AEs occurred in 8 patients (11.3%) in TATA sharp-falling class and 22 patients (6.6%) in TATA stable class (P = 0.260).

The PD-(L)1 inhibitors were discontinued due to AEs in 4 patients (6.9%) in SMM sharp-falling class and 5 patients (7.0%) in TATA sharp-falling class. Molecular targeted agents were discontinued in 5 patients (8.6%) because of AEs in SMM sharp-falling class, and 5 patients (7.0%) in TATA sharp-falling class. Dose interruptions of PD-(L)1 inhibitors were observed in 4 patients (6.9%) with SMM sharp-falling class and 2 patients (2.8%) with TATA sharp-falling class. Dose reduction or interruption of targeted agents was reported by 11 patients (19.0%) in the SMM sharp-falling class and 11 patients (15.5%) in the TATA sharp-falling class.

Treatment-emergent AEs were significantly higher in both SMM and TATA sharp-falling class (Table S9).

Table S1. Agents Administration Protocol

| **Agents** | **Targets** | **Administration** |
| --- | --- | --- |
| **Immune Checkpoints Inhibitors** | | |
| Atezolizumab, Tecentriq®, F. Hoffmann-La Roche AG, Basel, Switzerland | PD-L1 | 1200 mg once every 3 weeks, intravenous infusion |
| Sintilimab, Tyvyt®, Innovent Biologics, Inc., Suzhou, China | PD-1 | 200 mg once every 3 weeks, intravenous infusion |
| Camrelizumab, AiRuiKa®, Jiangsu Hengrui Medicine Co. Ltd, Suzhou, China | PD-1 | 200 mg once or 3mg/kg once every 3 weeks, intravenous infusion |
| Nivolumab, Opdivo®, Bristol-Myers Squibb Holdings Pharma, Ltd.Liability Company, New York, U.S.A. | PD-1 | 3 mg/kg once every 2 weeks, intravenous infusion |
| Pembrolizumab, Keytruda®, Merck Sharp& Dohme Corp., Kenilworth, N.J., U.S.A. | PD-1 | 200 mg once every 3 weeks, intravenous infusion |
| Tislelizumab, Baize’an®, BeiGene Ltd., Beijing, China | PD-1 | 200 mg once every 3 weeks, intravenous infusion |
| **Anti-VEGF antibody/TKIs** | | |
| Sorafenib, Nexavar®, Bayer AG Kaiser-Wilhelm-Allee, Leverkusen, Germany | VEGFR1–VEGFR3,  PDGFR, RAF kinase, KIT receptor | 400 mg BID |
| Lenvatinib, Lenvanix®, Eisai Inc., Japan | VEGFR1–VEGFR3, PDGFR, FGFR1–FGFR4, RET | 8 mg QD (for bodyweight <60 kg) or 12mg QD (for bodyweight ≥60 kg) |
| Bevacizumab, Avastin®, F. Hoffmann-La Roche AG, Basel, Switzerland | VEGFA | 15 mg/kg once every 3 weeks, intravenous infusion |
| Donafenib, Zepsun®, Suzhou Zelgen Biopharmaceuticals Co, Ltd., Suzhou, China | VEGFR, PDGFR, Raf/MEK/ERK kinase | 200 mg BID |
| Apatinib, Aitan®, Jiangsu Hengrui Medicine Co. Ltd, Lianyungang, China | VEGFR2, c-Kit, c-SRC | 250 mg QD |

Table S2. Number of patients treated with combination therapy

| **Therapy modality** | **Number** |
| --- | --- |
| PD-1 inhibitors plus anti-VEGF antibody | 57 (13.9) |
| PD-1 inhibitors plus TKIs | 331 (80.5) |
| PD-L1 inhibitors plus anti-VEGF antibody | 21 (5.1) |
| PD-L1 inhibitors plus TKIs | 2 (0.5) |

PD-1, programmed death-1; VEGF, vascular endothelial growth factor; TKIs, tyrosine kinase inhibitors; PD-L1, programmed death-ligand 1;

Table S3. LCGMM results of the model fitting process for SMM

A total of 411 patients with 2138 time-point measurements; LCGMM, latent class growth mixture models; SMM, skeletal muscle mass

| No. of latent classes | Polynomial degree | BIC | Patients Per class (%) | Mean posterior probabilities |
| --- | --- | --- | --- | --- |
| 1 | Linear | 16280.76 | 100 | - |
|  | Quadratic | 16148.65 | 100 | - |
|  | Cubic | 104371.16 | 100 | - |
| 2 | Linear | 16224.68 | 8.27/91.73 | 0.80/0.97 |
|  | **Quadratic** | **16134.11** | **14.11/85.89** | **0.80/0.87** |
|  | Cubic | 18724.55 | 100/0 | 1/0 |
| 3 | Linear | 16244.62 | 8.27/89.29/2.43 | 0.80/0.95/0.81 |
|  | Quadratic | 16161.86 | 19.22/78.83/1.95 | 0.79/0.79/0.90 |
|  | Cubic | 24563.36 | 0/0/100 | 0/ 0/1 |
| 4 | Linear | 16264.55 | 1.95/40.88/33.82/23.36 | 0.86/0.68/0.76/0.72 |
|  | Quadratic | 16191.63 | 10.71/14.36/65.94/9.00 | 0.77/0.67/0.72/0.57 |
|  | Cubic | NA | NA | NA |
| 5 | Linear | 16286.67 | 29.68/7.3/1.7/37.47/23.84 | 0.74/0.55/0.88/0.67/0.72 |
|  | Quadratic | 16215.99 | 54.99/3.65/7.3/10.71/23.36 | 0.62/0.77/0.62/0.58/0.57 |
|  | Cubic | 18878.35 | 0/100/0/0/0 | 0/1/0/0/0 |
| 6 | Linear | 16315.48 | 4.38/1.95/53.28/4.38/23.6/12.41 | 0.58/0.84/0.68/0.64/0.70/0.73 |
|  | Quadratic | 16237.53 | 4.87/24.09/5.11/23.6/28.47/13.87 | 0.79/0.53/0.74/0.52/0.60/0.56 |
|  | Cubic | 19258.35 | 0/0/0/100/0/0 | 0/0/0/1/0/0 |

Table S4. LCGMM results of the model fitting process for TATA

A total of 406 patients with 2056 time-point measurements; LCGMM, latent class growth mixture models; TATA, total adipose tissue area

| No. of latent classes | Polynomial degree | BIC | Patients Per class (%) | Mean posterior probabilities |
| --- | --- | --- | --- | --- |
| 1 | Linear | 21261.78 | 100 | - |
|  | Quadratic | 21173.37 | 100 | - |
|  | Cubic | 2276588.92 | 100 | - |
| 2 | Linear | 21228.38 | 10.84/89.16 | 0.83/0.91 |
|  | **Quadratic** | **21145.60** | **17.49/82.51** | **0.80/0.87** |
|  | Cubic | NA | NA | NA |
| 3 | Linear | 21208.54 | 12.81/27.34/59.85 | 0.79/0.72/0.78 |
|  | Quadratic | 21129.77 | 17.98/28.57/53.45 | 0.79/0.73/0.75 |
|  | Cubic | 33176.70 | 0/0/100 | 0/0/1.00 |
| 4 | Linear | 21232.83 | 12.56/9.36/73.15/4.93 | 0.80/0.58/0.81/0.76 |
|  | Quadratic | 21153.24 | 35.22/13.79/42.86/8.13 | 0.74/0.81/0.80/0.74 |
|  | Cubic | 24113.87 | 0/0/100/0 | 0/0/1.00/0 |
| 5 | Linear | 21259.44 | 5.67/28.08/62.56/1.48/2.22 | 0.87/0.71/0.81/0.56/0.85 |
|  | Quadratic | 21155.72 | 5.17/47.78/31.53/0.99/14.53 | 0.84/0.76/0.73/0.92/0.79 |
|  | Cubic | 30161.17 | 100/0/0/0/0 | 1.00/0/0/0/0 |
| 6 | Linear | 21289.77 | 7.14/18.47/24.63/20.69/7.14/21.92 | 0.84/0.71/0.64/0.61/0.84/0.58 |
|  | Quadratic | 21191.22 | 10.34/36.7/27.83/8.13/10.59/6.4 | 0.64/0.72/0.64/0.80/0.79/0.66 |
|  | Cubic | 38902.19 | 81.77/0/0/0/18.23/0 | 0.98/0/0/0/0.95/0 |

**Table S5. Baseline characteristics of** **the presence of cachexia** **during follow-up**

|  | **Pre-cachexia**  **(n = 299)** | **Cachexia**  **(n = 86)** | **Refractory cachexia**  **(n = 21)** | ***P* value** |
| --- | --- | --- | --- | --- |
| Median age (years)# | 57 (49-65) | 53 (49-62) | 54 (51-59) | 0.321 |
| Sex |  |  |  | 0.340 |
| Female | 40 (13.4) | 8 (9.3) | 1 (4.8) |  |
| Male | 259 (86.6) | 78 (90.7) | 20 (95.2) |  |
| ECOG PS |  |  |  | 0.456 |
| 0 | 229 (76.6) | 62 (72.1) | 14 (66.7) |  |
| 1 | 70 (23.4) | 24 (27.9) | 7 (33.3) |  |
| Etiology |  |  |  | 0.786 |
| HBV | 233 (77.9) | 67 (77.9) | 15 (71.4) |  |
| Others | 66 (22.1) | 19 (22.1) | 6 (28.6) |  |
| Cirrhosis |  |  |  | 0.554 |
| Absent | 94 (31.4) | 22 (25.6) | 7 (33.3) |  |
| Present | 205 (68.6) | 64 (74.4) | 14 (66.7) |  |
| Child-Pugh class |  |  |  | 0.910 |
| A | 264 (88.3) | 75 (87.2) | 19 (90.5) |  |
| B | 35 (11.7) | 11 (12.8) | 2 (9.5) |  |
| BCLC stage |  |  |  | 0.109 |
| A | 33 (11.0) | 9 (10.5) | 3 (14.3) |  |
| B | 100 (33.4) | 27 (31.4) | 1 (4.8) |  |
| C | 166 (55.5) | 50 (58.1) | 17 (81.0) |  |
| Up-to-seven criteria |  |  |  | 0.589 |
| Within | 97 (32.4) | 27 (31.4) | 9 (42.9) |  |
| Beyond | 202 (67.6) | 59 (68.6) | 12 (57.1) |  |
| Macroscopic vascular invasion |  |  |  | 0.110 |
| Absent | 183 (61.2) | 50 (58.1) | 8 (38.1) |  |
| Present | 116 (38.8) | 36 (41.9) | 13 (61.9) |  |
| Extrahepatic spread |  |  |  | 0.136 |
| Absent | 210 (70.2) | 52 (60.5) | 12 (57.1) |  |
| Present | 89 (29.8) | 34 (39.5) | 9 (42.9) |  |
| Serum AFP level |  |  |  | 0.729 |
| ≤400 | 177 (59.8) | 47 (55.3) | 13 (61.9) |  |
| >400 | 119 (40.2) | 38 (44.7) | 8 (38.1) |  |

Except where indicated, data are number (%). Chi-squared test or Fisher exact test for categorical variables were applied.

^#^ Data were continuous variables, expressed in median (interquartile range), and were compared by using the Mann-Whitney U test.

BMI, body mass index; ECOG PS, Eastern Cooperative Oncology Group performance status; BCLC, Barcelona Clinic Liver Cancer; HBV, hepatitis B virus; AFP, alpha-fetoprotein; SMM, skeletal muscle mass; TATA, total adipose tissue area;

^*^Five cases were excluded due to unmeasurable visceral adipose tissue.

Table S6. Treatment-emergent AE of the presence of cachexia during follow-up

|  | **Body composition^#^** | | |  |
| --- | --- | --- | --- | --- |
|  | **Pre-cachexia**  (n = 299) | **Cachexia**  (n = 86) | **Refractory cachexia**  (n = 21) | **P value** |
| Treatment-emergent adverse event | 73 (24.4) | 30 (34.9) | 12 (57.1) | 0.002 |
| Grade 3 or 4 event^*^ | 18 (6.0) | 7 (8.1) | 5 (23.8) | 0.010 |
| Discontinuation of anti-PD-(L)1 therapies | 14 (4.7) | 3 (3.5) | 3 (14.3) | 0.114 |
| Discontinuation of molecular targeted therapies | 15 (5.0) | 2 (2.3) | 4 (19.0) | 0.008 |
| Dose interruption of anti-PD-(L)1 therapies | 13 (4.3) | 2 (2.3) | 2 (9.5) | 0.324 |
| Dose reduction or interruption of molecular targeted therapies | 32 (10.7) | 12 (14.0) | 5 (23.8) | 0.170 |

Data are n (%). * Numbers represent the highest grades assigned. # Five cases were excluded due to unmeasurable visceral adipose tissue. AE, adverse events

Table S7. The results of joint modeling

|  | **HR** | **95%CI** | **P value** |
| --- | --- | --- | --- |
| **OS analyses** |  |  |  |
| SMM | 0.851 | 0.702-0.938 | <0.001 |
| TATA | 0.961 | 0.917-0.986 | <0.001 |
| **PFS analyses** |  |  |  |
| SMM | 0.987 | 0.983-0.991 | <0.001 |
| TATA | 0.998 | 0.997-0.999 | <0.001 |

SMM, skeletal muscle mass; TATA, total adipose tissue area;

**Figure S1. Flowchart of the study.**

Five cases were excluded due to unmeasurable visceral adipose tissue for total adipose tissue area analysis (n = 406). HCC, hepatocellular carcinoma; PD-(L)1 inhibitors, programmed death-(ligand)1; anti-VEGF antibody/TKIs, anti-vascular endothelial growth factor antibody/tyrosine kinase inhibitors; ECOG, Eastern Cooperative Oncology Group; CT, computed tomography;


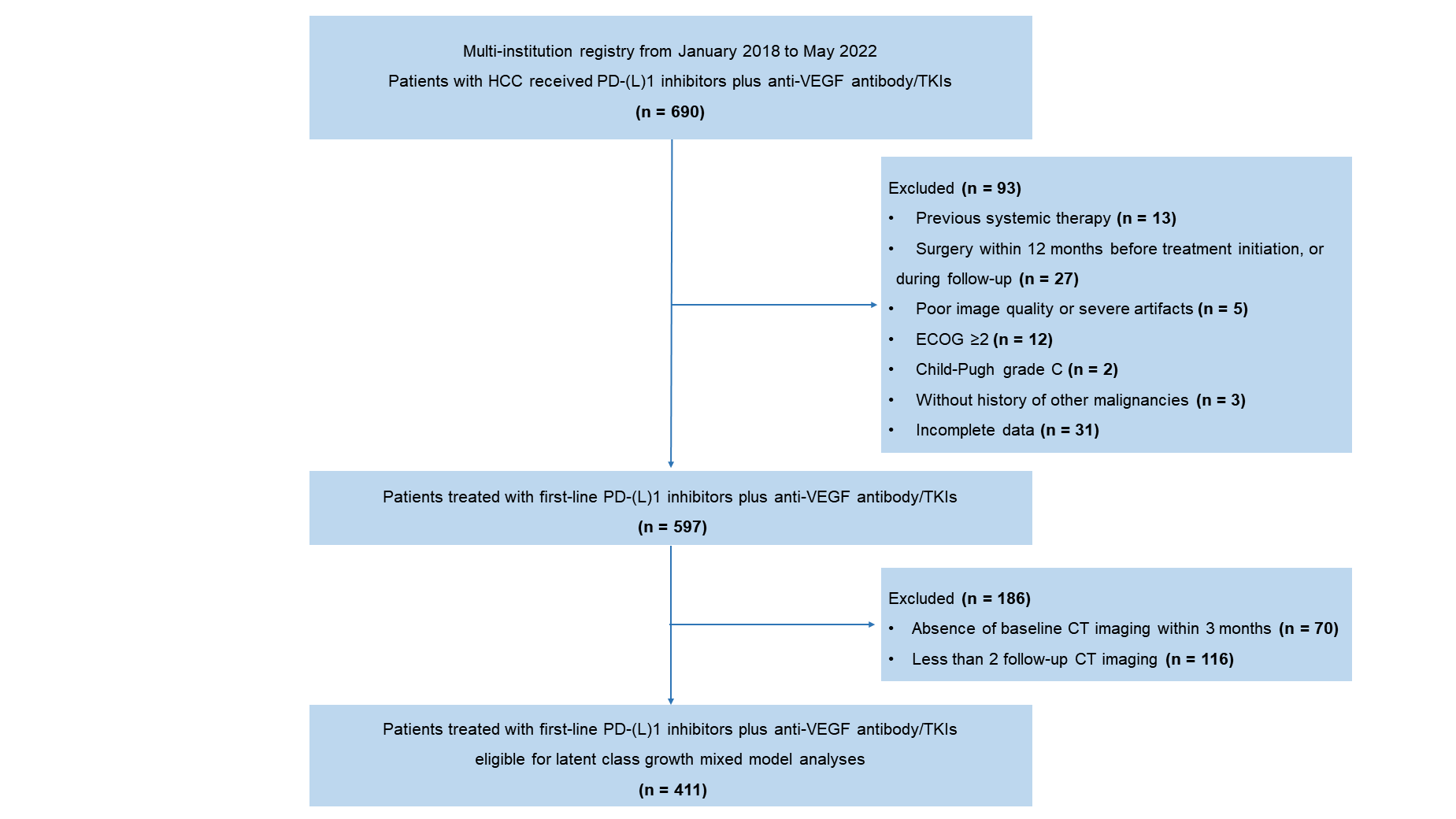


**Figure S2. Subgroup analysis of PFS for longitudinal SMM trajectory.**

PFS, progression-free survival; SMM, skeletal muscle mass; HR, hazard ratio; CI, confidence interval; BMI, body mass index; ECOG, Eastern Cooperative Oncology Group; AFP, alpha-fetoprotein; BCLC, Barcelona Clinic Liver Cancer;


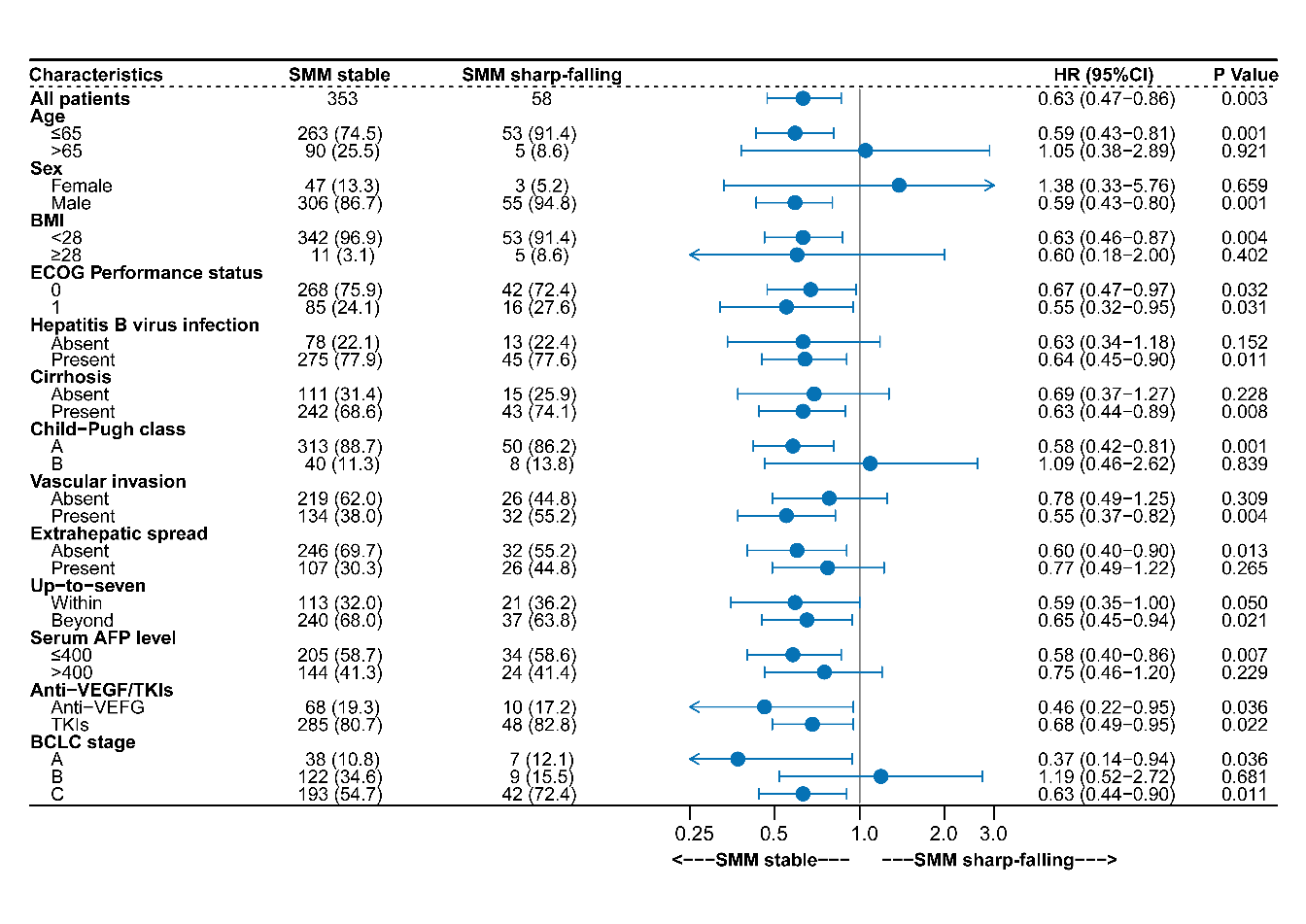


**Figure S3. Subgroup analysis of PFS for longitudinal TATA trajectory.**

PFS, progression-free survival; TATA, total adipose tissue area; HR, hazard ratio; CI, confidence interval; BMI, body mass index; ECOG, Eastern Cooperative Oncology Group; AFP, alpha-fetoprotein; BCLC, Barcelona Clinic Liver Cancer;


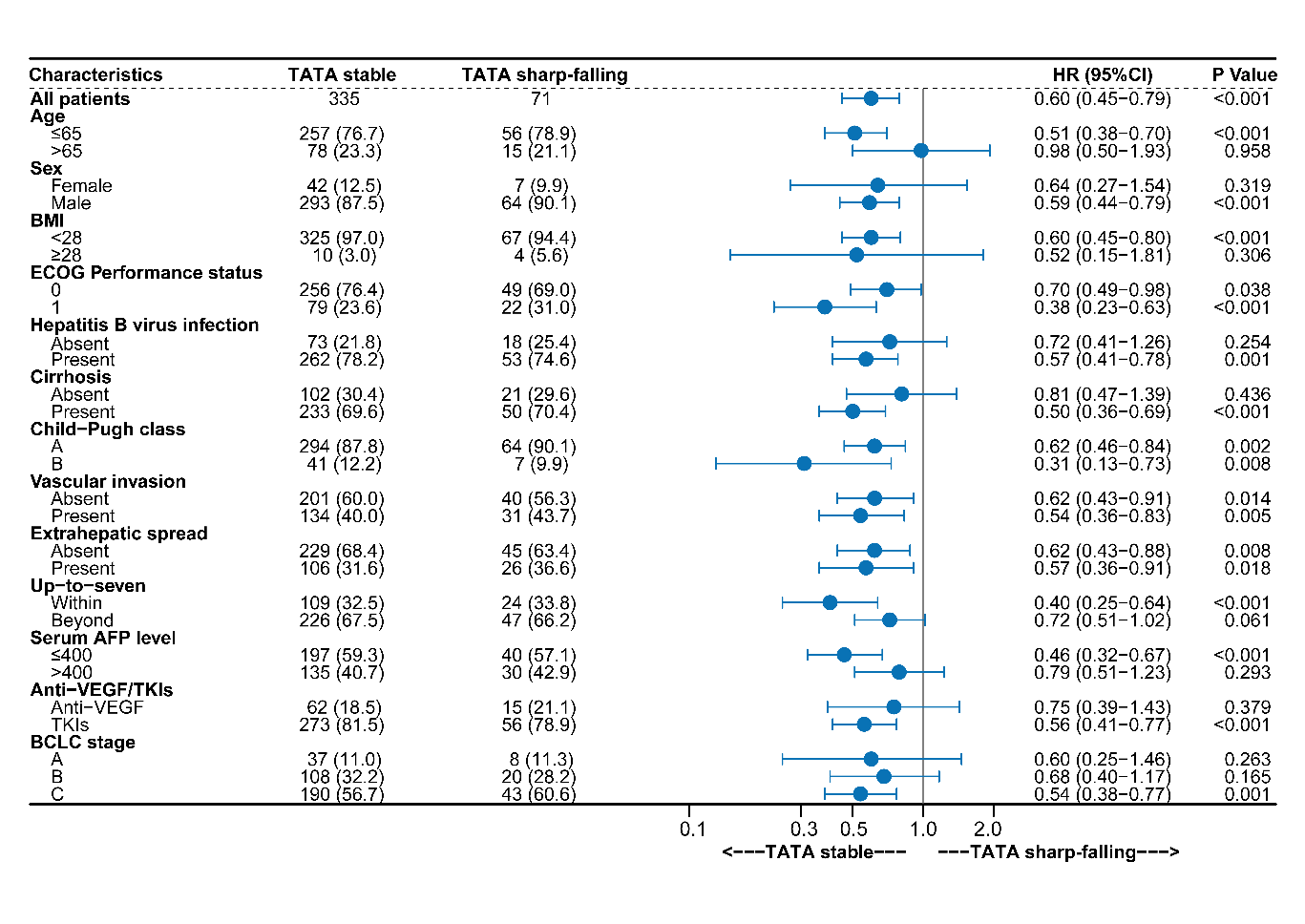


**Figure S4. Heatmap of the correlation between clinical variables and longitudinal body composition trajectory classes in HCC patients.**

BCLC, Barcelona Clinic Liver Cancer staging system; ECOG, Eastern Cooperative Oncology Group; AFP, alpha-fetoprotein; SMM, skeletal muscle mass; TATA, total adipose tissue area; BMI, body mass index; TATI, total adipose tissue index.


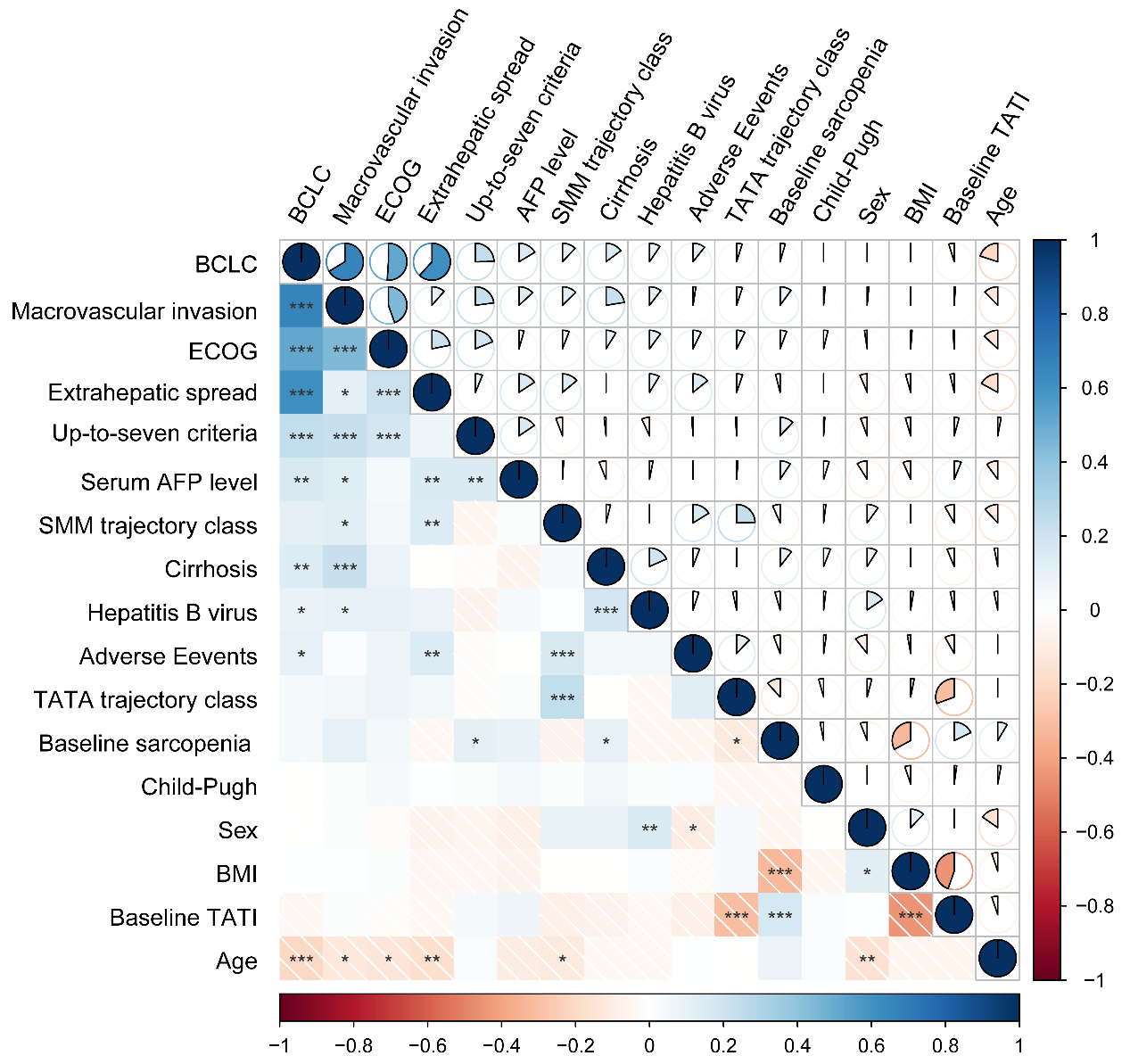


**Figure S5. Venn diagram of longitudinal SMM and baseline skeletal muscle (sarcopenia/non-sarcopenia) distributions.**

Baseline sarcopenia was determined based on previous study (L3 skeletal muscle index, 36.2 cm2/m2 for men and 29.6 cm2/m2 for women).^2^ SMM, skeletal muscle mass;

**
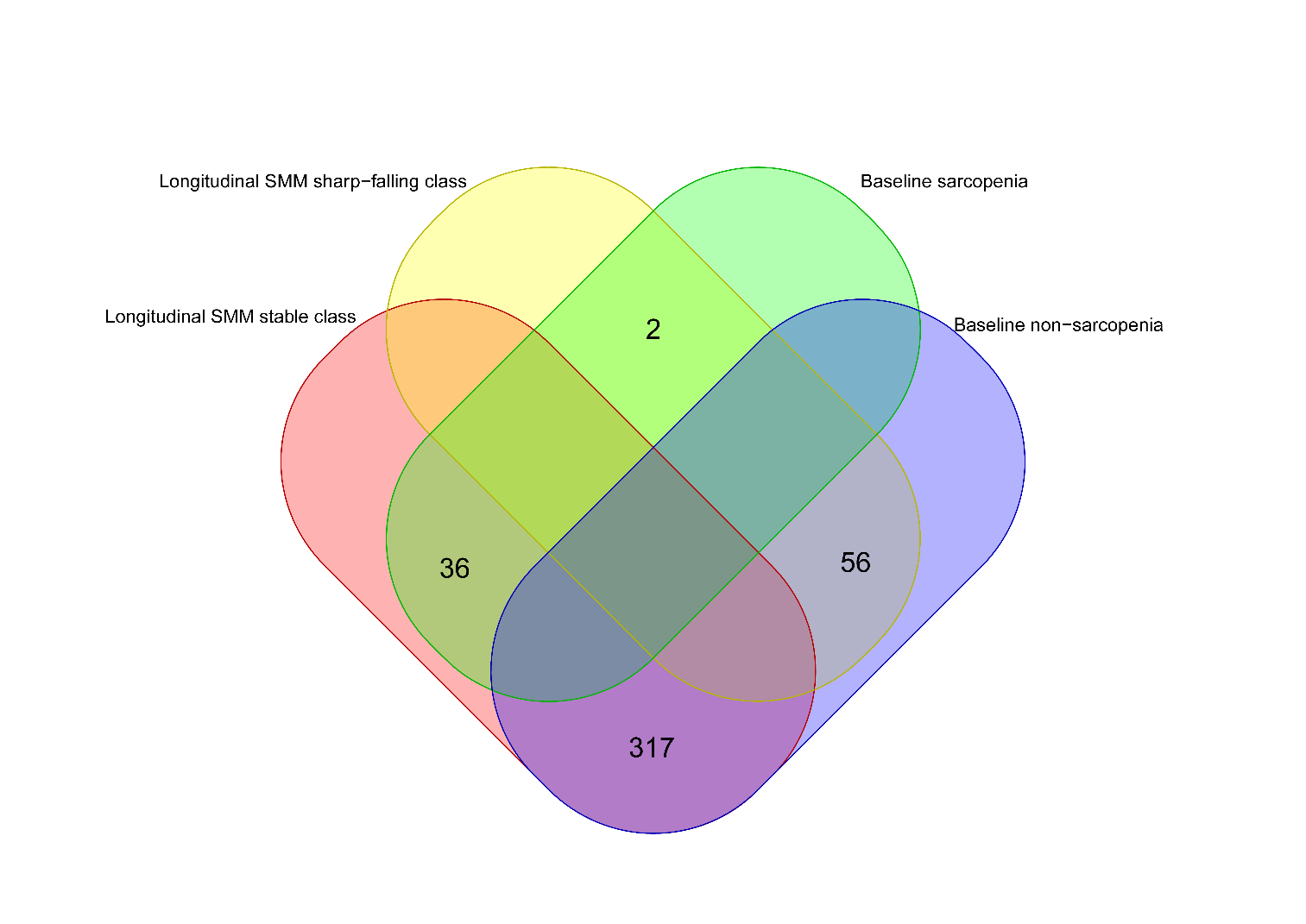
**

**Figure S6. Venn diagram of longitudinal TATA and baseline TATI distributions.**

Patients with baseline total adipose tissue area were dichotomized into 2 groups: sex-specific high (above median) TATI and low TATI (equal to or below median) groups. TATA, total adipose tissue area; TATI, total adipose tissue index;


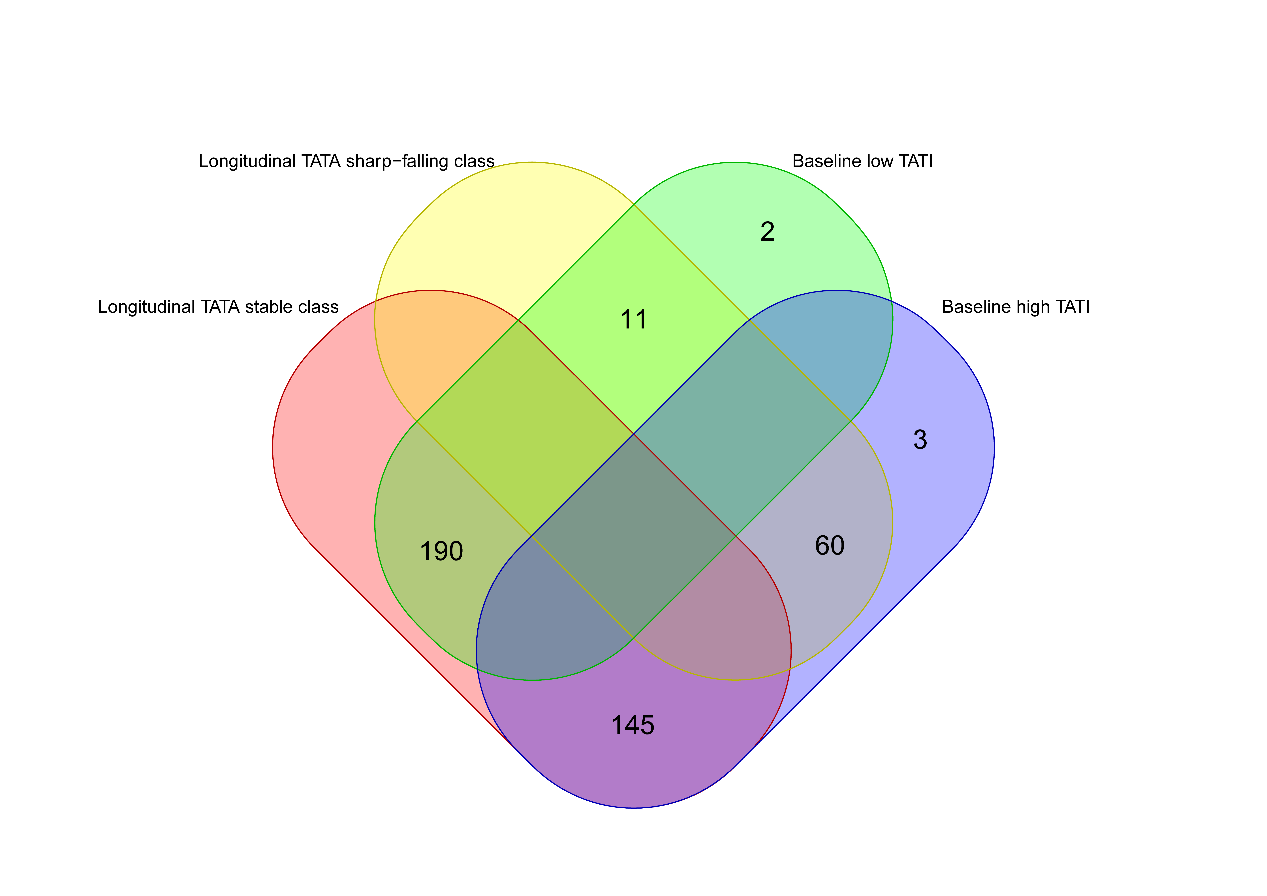


**Figure S7. Venn diagram of longitudinal SMM and TATA distributions**

SMM, skeletal muscle mass; TATA, total adipose tissue area;


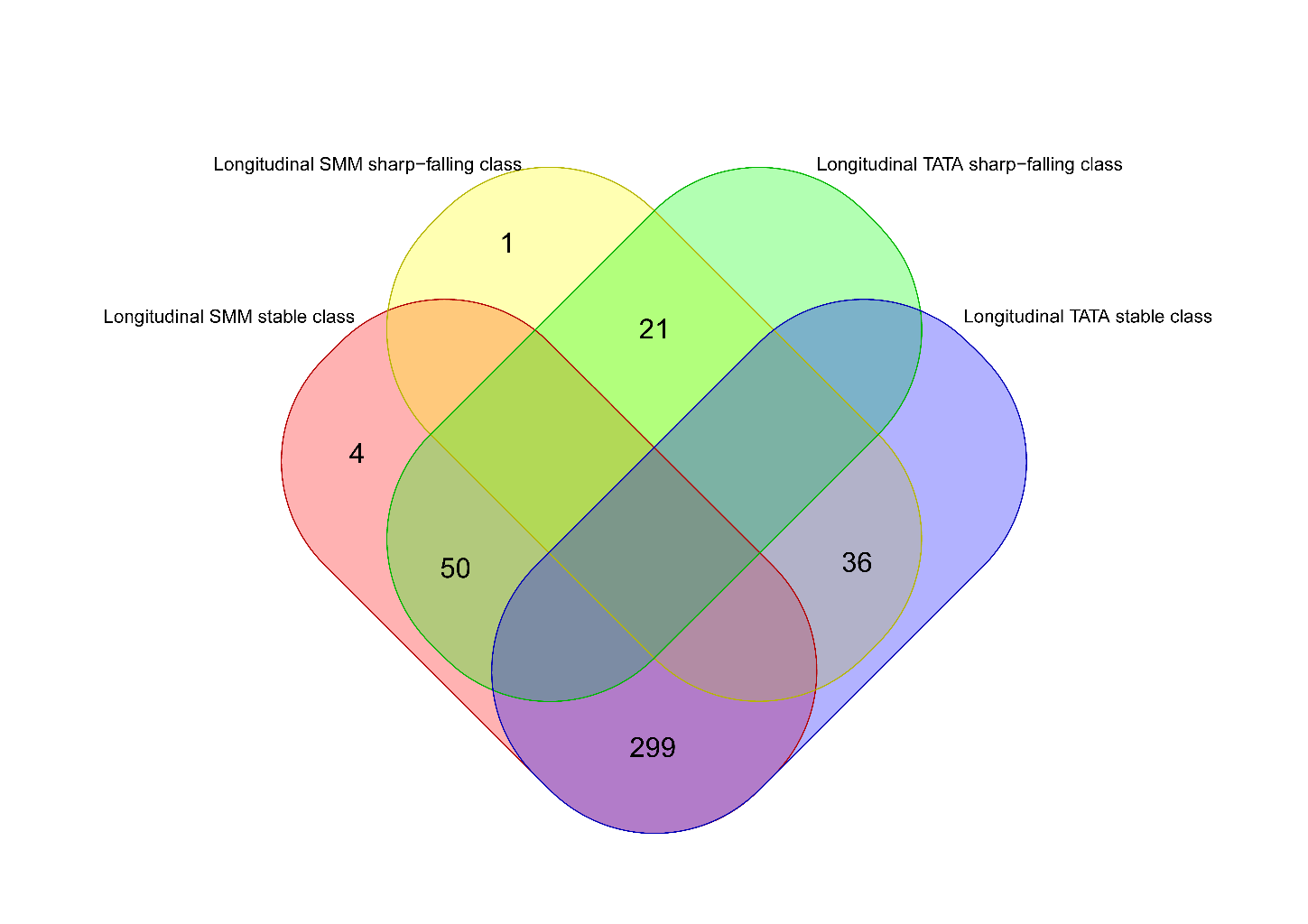


**Figure S8. The relative importance of each risk factor for OS.**

A) Baseline risk factors plus SMM trajectory classes; B) Baseline risk factors plus TATA trajectory classes; C) Baseline risk factors plus SMM trajectory classes and TATA trajectory classes; D) Baseline risk factors plus combined trajectory classes; OS, overall survival; SMM, skeletal muscle mass; TATA, total adipose tissue area;


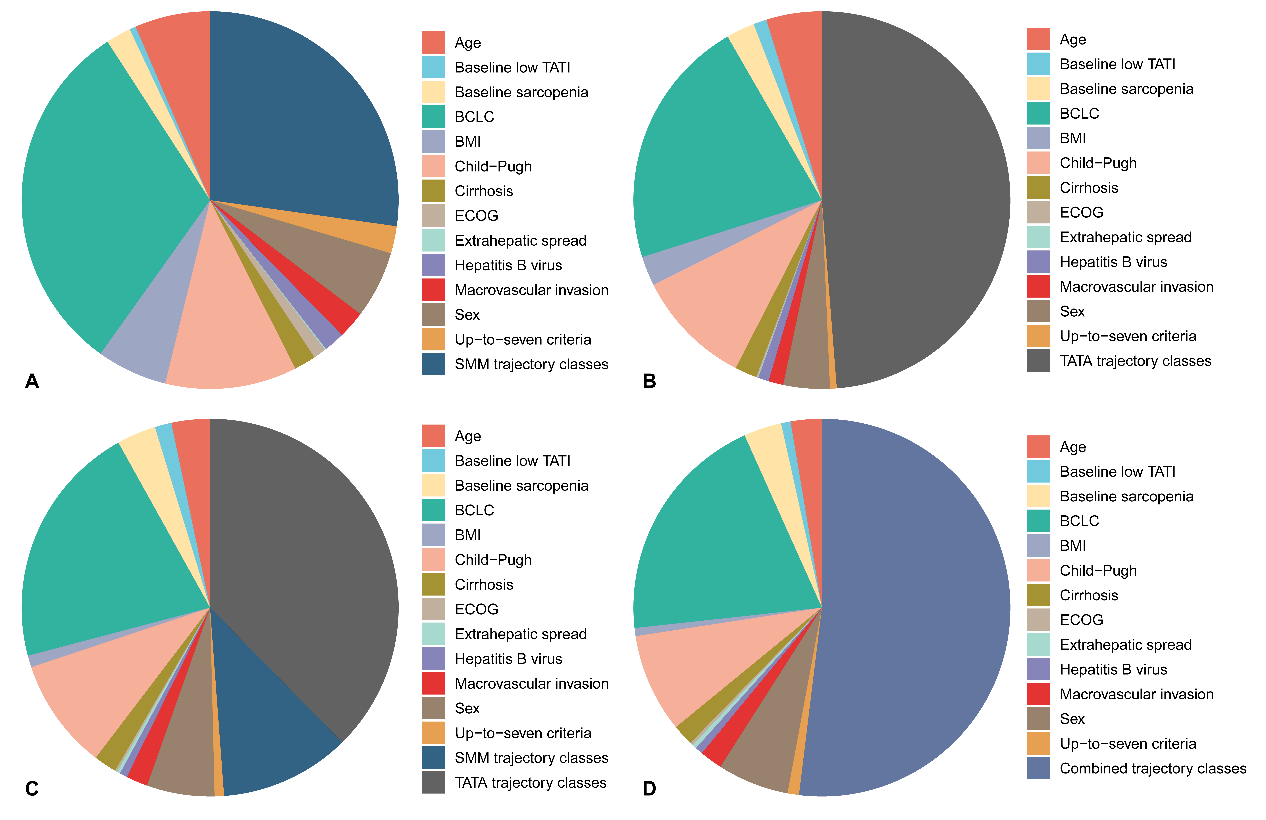


**Figure S9. The relative importance of each risk factor for PFS.**

A) Baseline risk factors plus SMM trajectory classes; B) Baseline risk factors plus TATA trajectory classes; C) Baseline risk factors plus SMM trajectory classes and TATA trajectory classes; D) Baseline risk factors plus combined trajectory classes; PFS, progression-free survival; SMM, skeletal muscle mass; TATA, total adipose tissue area;


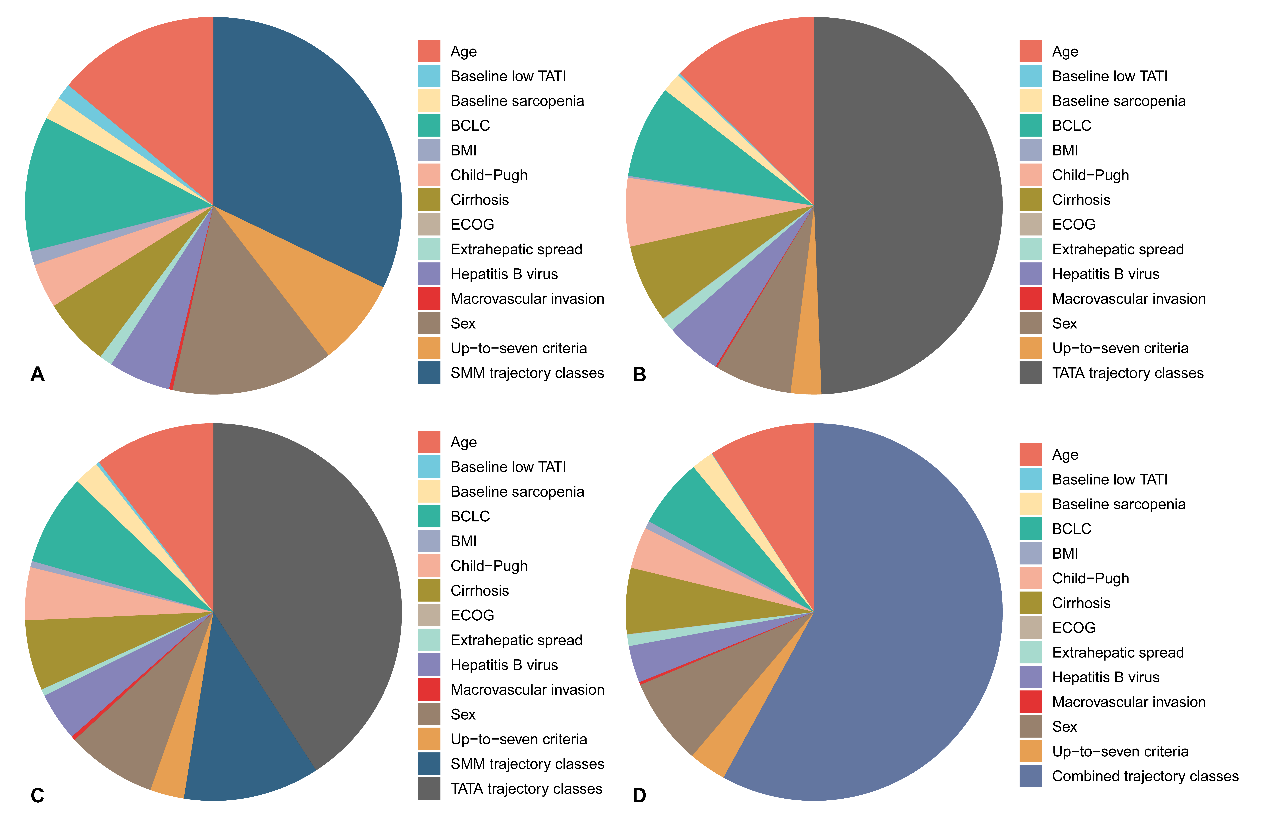


**Reference**

1. Ibrahim JG, Chu H, Chen LM. Basic concepts and methods for joint models of longitudinal and survival data. *Journal of clinical oncology : official journal of the American Society of Clinical Oncology*. Jun 1 2010;28(16):2796-801.

2. Fujiwara N, Nakagawa H, Kudo Y, et al. Sarcopenia, intramuscular fat deposition, and visceral adiposity independently predict the outcomes of hepatocellular carcinoma. *Journal of hepatology*. Jul 2015;63(1):131-40.
